# Antibiotic-Loaded Boron Nitride Nanoconjugate with Strong Performance against Planktonic Bacteria and Biofilms

**DOI:** 10.1021/acsabm.3c00247

**Published:** 2023-07-20

**Authors:** Jian Zhang, Nisha Neupane, Puspa Raj Dahal, Shadi Rahimi, Zhejian Cao, Santosh Pandit, Ivan Mijakovic

**Affiliations:** † Systems and Synthetic Biology Division, Department of Life Sciences, Chalmers University of Technology, SE-412 96 Gothenburg, Sweden; ‡ Department of Microbiology, Tri-Chandra Multiple College, Tribhuvan University, 44600 Kathmandu, Nepal; § The Novo Nordisk Foundation, Center for Biosustainability, Technical University of Denmark, DK-2800 Kogens Lyngby, Denmark

**Keywords:** boron nitride, polydopamine, gentamicin, antibacterial, antibiofilm

## Abstract

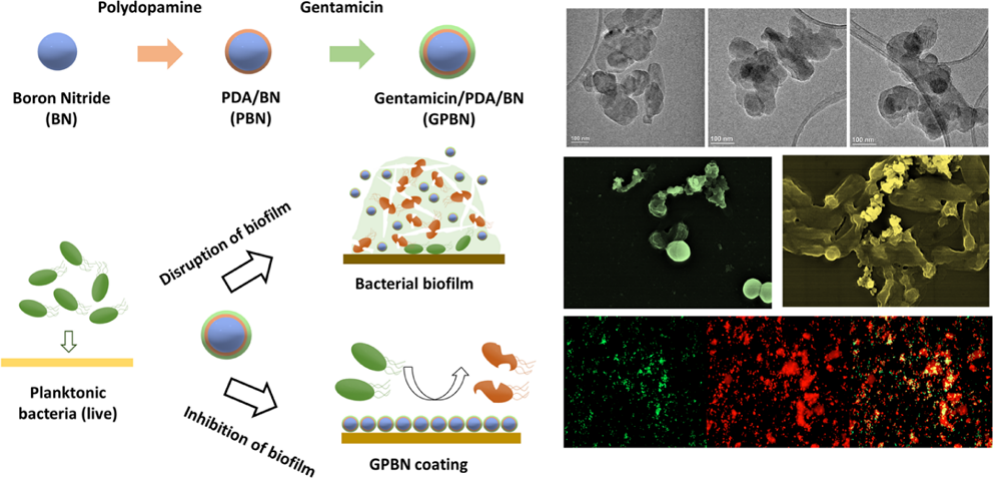

Protecting surfaces from biofilm formation presents a significant challenge in the biomedical field. The utilization of antimicrobial component-conjugated nanoparticles is becoming an attractive strategy against infectious biofilms. Boron nitride (BN) nanomaterials have a unique biomedical application value due to their excellent biocompatibility. Here, we developed antibiotic-loaded BN nanoconjugates to combat bacterial biofilms. Antibiofilm testing included two types of pathogens, *Staphylococcus aureus* and *Escherichia coli*. Gentamicin was loaded on polydopamine-modified BN nanoparticles (GPBN) to construct a nanoconjugate, which was very effective in killing *E. coli* and *S. aureus* planktonic cells. GPBN exhibited equally strong capacity for biofilm destruction, tested on preformed biofilms. A 24 h treatment with the nanoconjugate reduced cell viability by more than 90%. Our results suggest that GPBN adheres to the surface of the biofilm, penetrates inside the biofilm matrix, and finally deactivates the cells. Interestingly, the GPBN coatings also strongly inhibited the formation of bacterial biofilms. Based on these results, we suggest that GPBN could serve as an effective means for treating biofilm-associated infections and as coatings for biofilm prevention.

## 1 Introduction

Biofilms are considered a key culprit for failure of medical devices and biomedical implants.^1^ Biofilm formation is one of the major mechanisms of resistance to known antibiotics. Compared with planktonic bacteria, bacterial cells in biofilms are highly resistant to antibiotics. Microbial cells in biofilms adhere to each other to form stacked microcolonies, which are surrounded and impregnated by a self-produced extracellular polymer matrix (EPS). EPS can slow the penetration of antimicrobials into biofilms and enzymatic components of the EPS can trigger modification or inactivation of antimicrobials.^2,3^ In recent years, nanomaterials have shown considerable potential against biofilms because of their intrinsic antibacterial activity, the ability to serve as drug delivery vehicles, or their potential to circumvent bacterial resistance mechanisms.^4,5^

Conventional antibiotics are often ineffective in treating biofilms because bacteria within biofilms have evolved multiple mechanisms to evade antibiotic attack. Therefore, effective therapeutic strategies with novel anti-biofilm modes of action are urgently needed. In this context, nanomedicine has gained great attention and has shown promise in the prevention and elimination of bacterial biofilms.^6^ For example, some nanomaterials with enzyme-like catalytic activity can kill bacteria and destroy the biofilm matrix by catalyzing the production of reactive oxygen species.^7−9^ The photophysical and photochemical properties of nanomaterials have been exploited for photothermal and photodynamic therapy of biofilms.^10,11^ Notably, drug nanocarriers have been widely exploited to modulate the pharmacokinetics and efficacy of drugs to maximize their antibacterial potential and minimize adverse effects on normal tissues or cells.^5,12^ For example, the graphene family has been widely studied as an innovative drug nanocarrier for a variety of agents, including anticancer drugs, insoluble drugs, antibiotics, antibodies, peptides, DNA, RNA, and genes. These substances can be attached to the graphene surface through non-covalent interactions or covalent bonding.^13,14^ In addition to the assembly of one drug into nanocarriers, the combination drug therapy has advantages in biofilm eradication.^15,16^ Combination therapy of antibacterial drugs can exert synergistic effects by acting on different targets or different pathways of bacteria. This approach has many benefits such as overcoming multi-drug resistance, improving drug efficacy, and reducing side effects. At present, nanocarriers are also widely used to deliver multiple combined drugs to improve bioavailability and meet diverse therapeutic needs.^17,18^ In addition to loading drugs onto nanocarriers, recently, a dynamic covalent bonding strategy was developed and applied to drug delivery.^16^ This strategy offers possibilities to generate nanostructures using different building blocks (drugs, lipids, polymer precursors, etc.).^19−21^ These reversible dynamic covalently bonded drug delivery systems have good chemical structure and high drug loading capacity, and under a specific microenvironment, the dynamic balance can be broken so that the drug can be released in an on-demand manner. Hence, the development of nanomaterials and nanotechnology has great potential for drug delivery and targeted treatment of bacterial infections.

Hexagonal boron nitride (h-BN) consists of sp^2^-hybridized, alternating boron and nitrogen atoms, showing a honeycomb structure similar to that of graphene.^22^ BN nanomaterials (nanoparticles, nanotubes, nanosheets, etc.) have excellent chemical stability, thermal stability, high thermal conductivity, and excellent electrical insulation. In recent years, BN nanomaterials are being used for different application fields due to their unique properties. BN nanomaterials have been used in optoelectronic devices, hydrogen storage devices, insulating substrates, and lubricating coatings.^23−26^ BN nanostructures have been widely explored and used as a biocompatible material in various biological applications.^27−31^ Functionalization of BN nanomaterials is necessary for biological applications such as cancer therapy and drug delivery.^32−37^ For example, Sukhorukova et al. reported that BN nanoparticles with a petal-like surface can effectively load anticancer drugs (such as doxorubicin, DOX), and DOX-loaded BN nanoparticles effectively release DOX into the cytoplasm and nucleus, leading to tumor IAR-6-1 cell death.^38^ In addition, antibacterial activities based on BN and hybrid BN nanomaterials have also been studied.^39−42^ Maria et al. reported that aqueous dispersion of polymer-coated BN nanotubes especially with polyethyleneimine exhibit strong antibacterial effects against *Escherichia coli* (*E. coli*) and *Staphylococcus aureus* (*S. aureus*).^43^ Guan et al. developed a novel functionalized BN material as a carrier to deliver volatile tea tree oil (TTO) to enhance antibacterial activity. Polysaccharide (carboxymethylcellulose, CMC)- and soy protein isolate (SPI)-functionalized BN nanosheets (CMC-SPI-BNNS) achieved a high loading capacity of TTO. The antibacterial activity of TTO-loaded CMC-SPI-BNNS was improved, and the protective effect on the active ingredient was excellent.^44^ Gudz et al. reported the antibacterial study of BN nanoparticles grown by the chemical vapor deposition method. They demonstrated that antibiotic-resistant *E. coli* were killed by physical contact destruction when exposed to nanosheet- and nanoneedle-structured BN nanoparticle films.^45^ This BN nanoparticle exhibited high antibiotic-loading capacity, providing bactericidal protection against a variety of pathogenic strains.^45,46^ BN materials are rarely used in antibacterial research. Moreover, majority of available research studies are mainly focused on examining their activity against planktonic bacteria. However, available data on the antimicrobial properties of BN against biofilms are rather scarce.

Recently, biopolymers such as polydopamine (PDA) have attracted extensive attention as effective drug carriers.^47^ PDA has been significantly developed in the biomedical field based on its biocompatibility, biodegradability, and nontoxicity.^48^ The excellent adhesive properties of PDA and its ability to perfectly immobilize various biomolecules and metal ions facilitate the development of appropriate implant surface modifications.^49,50^ In recent years, several combinations of different substrates and functionalized biomolecules such as liposome have been efficiently exploited with PDA coatings.^51,52^ For example, dexamethasone-loaded liposomes were modified on PDA coatings to construct porous coatings, which exhibited antibacterial activity against *E. coli*, *S. aureus*, *Porphyromonas gingivalis,* and *Streptococcus mutans*.^53,54^ PDA coating was observed to enhance the dispersion of BN nanotubes without exhibiting cytotoxicity.^55^ PDA, a mussel biomimetic material, can be obtained by self-polymerization of dopamine in a weakly alkaline environment. The formation of PDA is based on the equilibrium movement mechanism by which catechol in dopamine is oxidized to quinones by alkaline pH-induced oxidation to eventually produce polydopamine.^56^ When BN nanoparticles were added to an aqueous solution of dopamine, significant adhesion was expected due to the strong interactions between the aromatic molecules of dopamine and BN through π–π stacking forces and van der Waals interactions.^57,58^ In addition, the polydopamine adsorbed on the surface of the material can act as a reaction bridge to further react with reagents containing nucleophilic groups through Michael addition or Schiff base reaction.^59^ The reactive catechol groups exposed on PDA can be covalently grafted with amine groups. This facile and efficient covalent approach is beneficial to construct nanoconjugates loaded with the aminoglycoside antibiotic.^60−62^

Gentamicin is an aminoglycoside antibiotic commonly used to treat a variety of bacterial infections such as those of the respiratory and urinary tracts, blood, bone, and soft tissues due to its low cost, stability, broad-spectrum action, and effectiveness against major pathogens infect.^63^ However, gentamicin can have potential side effects such as nephrotoxicity and ototoxicity, particularly when given in high doses or for extended periods.^64^ To address the issues, incorporation of gentamicin into nanomaterials could mitigate side effects by releasing the drug in a controlled manner over time, reducing the need for frequent dosing and reducing the risk of toxicity.^65^ In this work, we attempted to develop antibiotic-loaded BN nanoconjugates with strong anti-biofilm potential. PDA-functionalized BN nanoparticles (PBN) were covalently conjugated with gentamicin to obtain gentamicin-loaded BN (GPBN) nanoconjugates, which were evaluated against the anti-biofilm activity of two pathogens *S. aureus* and *E. coli*. Once exposed to the planktonic bacteria or biofilms formed by *S. aureus* or *E. coli* cells, the GPBN result in strong bactericidal efficiency and significant disruption of bacterial biofilms. In addition, based on PDA’s strong adhesion ability, GPBN can be applied to any surface as a coating to offer a practical and effective solution on surfaces that are prone to biofilm formation. Our results suggest that antibiotic-loaded BN nanoconjugates can be a very effective approach of combating biofilms such as for antimicrobial protection of biomedical devices or as an alternative treatment option for biofilm infection.

## 2 Experimental Section

### 2.1 Bacterial Strains, Chemicals, and Materials

BN ultra-fine powder (average particle size: 70 nm; specific surface area: 20 m^2^/g) was obtained from the Graphene supermarket (USA). Dopamine hydrochloride and gentamicin sulfate were purchased from Sigma-Aldrich (Sweden). *E. coli* (*E. coli*) UTI89 and *S. aureus* (*S. aureus*) CCUG10778 were purchased from the Culture Collection University Of Gothenburg.

### 2.2 Preparation of PBN and GPBN Nanoconjugate

BN ultra-fine powder (400 mg) was dispersed in 200 mL of Tris buffer (10 mM, pH 8.5) by ultrasound for 2 h. Then, dopamine hydrochloride (400 mg) was added, and the mixture solution was mechanically stirred for 6 h at room temperature. The PBN was separated through centrifugation, washed with deionized water, and freeze-dried for further use. Subsequently, PBN (300 mg) was dispersed in sodium bicarbonate (NaHCO_3_) buffer (10 mM, pH 8.5) and gentamicin sulfate (300 mg) was added. The suspension was stirred for 48 h. The gentamicin-loaded GPBN was collected through centrifugation, washed with deionized water, and freeze-dried.

### 2.3 Characterizations

Attenuated total reflectance Fourier-transform infrared (ATR–FTIR) spectra were recorded on a Bruker’s Alpha spectrometer, utilizing a diamond crystal as the refractive element. The spectra were acquired by averaging 256 scans at a resolution of 4 cm^–1^. Powder X-ray diffraction (PXRD) was performed using a Bruker XRD D8 ADVANCE with Cu Kα1 radiation. Data were collected with 2θ from 20 to 70°. Raman spectra were obtained using a WITec alpha300 Raman spectrometer equipped with a 100× objective lens. A 532 nm wavelength laser and a 600 g/mm grating were employed for excitation. Spectra were acquired in the range of 500–3000 cm^–1^ with a 0.5 s integration time. The surface chemistry was analyzed using an X-ray photoelectron spectroscopy (XPS) instrument (PHI VersaProbe III). Monochromatized Al Kα X-ray (1486.6 eV) was employed to collect the spectra. Spectra were obtained using CasaXPS software. Dynamic light scattering (DLS) (Zetasizer Nano ZS, Japan) was used to study the size distribution and zeta potential of BN nanoconjugates. The shape and size of BN nanoconjugates was examined by scanning electron microscopy (SEM, JEOL JSM 6301F) under an acceleration voltage of 5 kV. Transmission electron microscopy (TEM) was carried out using a FEI Tecnai T20 transmission electron microscope under an acceleration voltage of 200 kV.

### 2.4 Gentamicin Loading and Release

Gentamicin loading and release were conducted by complexing gentamicin with the *o*-phthaldialdehyde reagent as reported before.^66^ Different batches were prepared by altering the concentration of gentamicin (50, 100, 200, 500 μg/mL) in the reaction mixture while maintaining a constant concentration of PBN (1000 μg/mL). The loading content of gentamicin was determined by calculating the amount of residual gentamicin in the solution using the following formula.
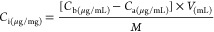
where *C*_i(μg/mg)_ is the amount of gentamicin loaded on PBN (μg/mg of PBN weight), *C*_b(μg/mL)_ is the concentration of gentamicin in solution before loading on PBN (μg/mL), *C*_a(μg/mL)_ is the concentration of gentamicin in solution after loading on PBN (μg/mL), *V*_(mL)_ is the volume of the solution with PBN (mL), and *M* is the weight of PBN (mg).

Alternatively, gentamicin release from GPBN nanoconjugates was tested through the dialysis method. For this purpose, a dialysis tube was filled with 3 mL of 1 mg/mL of the GPBN nanoconjugates in 20 mL of PBS buffer at pH 7.4. At predetermined time intervals, 1 mL of release medium was taken and replaced by the same quantity of fresh buffer. The released gentamicin concentrations were determined as described above.

### 2.5 Time Kill Assay against Planktonic Cells

The bactericidal efficiency of GPBN nanoconjugates was tested against planktonic cells. A growth medium containing sterile deionized water (control), 500 μg/mL of PBN (PBN_500_), 250, 500, and 1000 μg/mL of GPBN nanoconjugates (GPBN_250_, GPBN_500_, and GPBN_1000_) were inoculated with an overnight culture of inoculum containing 2–5 × 10^7^ cfu/mL bacterial cells.

All samples were incubated at 37 °C with continuous agitation for 24 h. At time points of 0, 3, 6, and 24 h, to determine bacterial viability, 100 μL of culture was extracted from each sample. The collected samples were then serially diluted in 0.89% NaCl solution and plated on agar plates. Following an incubation period of 24 h at 37 °C, the number of colonies was counted.

### 2.6 BN Nanoconjugate Efficiency against Preformed Biofilms

The biofilm disruption efficiency of GPBN nanoconjugate exposure was analyzed using three different methods: (1) by examining bacterial viability, (2) by observing cells under SEM, and (3) by live/dead staining assay. An overnight culture of inoculum containing 2–5 × 10^6^ cfu/mL bacterial cells was inoculated on top of a cover glass and incubated statically at 37 °C to form a biofilm. After 24 h, the old culture medium was replaced with fresh medium containing sterile water (control) or various concentrations of GPBN (GPBN_250_, GPBN_500_, and GPBN_1000_). Samples were incubated for further 24 h. The biofilms on the coatings were collected using 5 mL of 0.89% NaCl and subsequently homogenized through sonication. The homogenized biofilm suspension was then serially diluted in 0.89% NaCl and plated on plates. Following incubation at 37 °C for 24 h, the number of colonies was counted. Live/dead staining assay and SEM microscopy of morphology change of biofilms were performed as previously described.^67^

### 2.7 Biofilm Formation Assay for BN Nanoconjugate Coatings

Clean glass surfaces were coated with different concentrations of GPBN (GPBN_250_, GPBN_500_, and GPBN_1000_) using drop casting. PBN_500_ served as a control. 400 μL of dispersed GPBN and PBN solutions were placed on the glass surface and dried completely. The biofilm inhibition efficiency of the BN nanoconjugate coating was evaluated against *S. aureus* and *E. coli*. A 400 μL bacterial inoculum (2–5 × 10^6^ cfu/mL) from an overnight culture was inoculated on the coated surface and incubated at 37 °C for 24 h without agitation to form a biofilm. After 24 h, the biofilms were washed with sterile water to remove loosely attached or free bacterial cells and collected using 5 mL of 0.89% NaCl and subsequently homogenized through sonication. The resulting homogenized biofilm suspension was then serially diluted in 0.89% NaCl and plated on plates. Following incubation at 37 °C for 24 h, the number of colonies was counted. Live/dead staining assay of biofilms grown on the BN nanoconjugate-coated surfaces was performed as previously described.^67^

### 2.8 Biocompatibility for BN Nanoconjugates

The Huh 7 human hepatocyte cell line was used in this study. Cells were seeded on 96-well plates at a density of 2 × 10^4^ cells per well and incubated for 24 h, followed by treatment with different concentrations of BN, PBN, and GPBN nanoconjugates for 24 h. Subsequently, cells were incubated with medium containing 1× alamarBlue (Thermo Scientific) staining solution for a duration of 3 h. The signals emitted by the cells were detected using a FLUOStar Omega plate reader. The results obtained were normalized to the medium control.

### 2.9 Statistical Analysis

All measurements were conducted in triplicate, and the data are presented as the mean ± standard deviation. One-way analysis of variance followed by post hoc multiple comparison (Tukey) test was employed for statistical analysis. A *P*-value of less than 0.05 was considered statistically significant.

## 3 Results and Discussion

### 3.1 Gentamicin and PDA Were Functionalized on BN Nanoparticles to Construct GPBN Nanoconjugates

**Figure 1:**
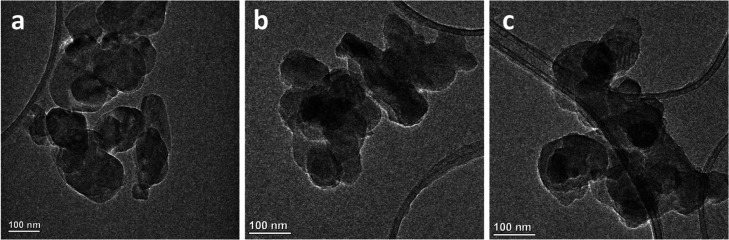
TEM images of BN (a), PBN (b), and GPBN (c).

**Figure 2:**
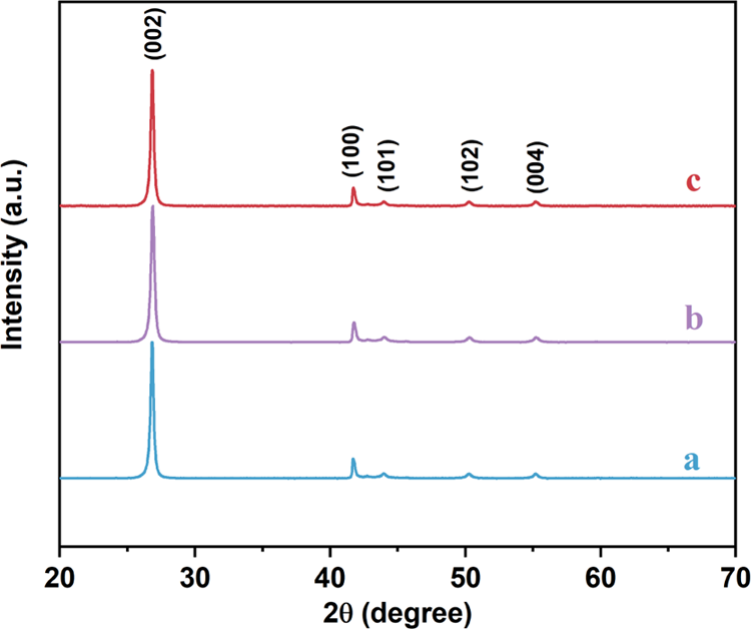
XRD patterns of BN (a), PBN (b), and GPBN (c).

The surface morphologies of BN PBN, and GPBN nanoconjugates were studied by SEM and TEM. SEM images of BN, PBN, and GPBN are displayed in Figure S1a–c. It showed that the size of the BN nanoparticles was homogeneous, and the particles ranged in size between 50 and 150 nm. After polydopamine functionalization and gentamicin loading, the morphology of PBN and GPBN did not change significantly since the polydopamine and gentamicin formed a very thin film on the surface of BN nanoparticles. The visual color of BN, PBN, and GPBN nanoconjugates are shown in the inset of Figure S1a–c. The color of the BN nanoparticles changes from white to gray-blue after PDA modification and gentamicin loading. The coloration of the samples is evidenced by the formation of PBN and GPBN nanoconjugates. Energy-dispersive spectrometry elemental mapping of GPBN (Figure S1) provided confirmation of the presence of C, B, N, and O elements. Moreover, the elements are evenly distributed within the nanoconjugates. The morphology difference of BN, PBN, and GPBN could be observed on TEM images. Evidently, Figure 1a shows that the pristine BN nanoparticles have smooth edges and high transparency. However, PBN and GPBN were slightly grayish black, the transparency was reduced, and the edges become rough, as shown in Figure 1b,c, indicating that the BN nanoparticles were successfully functionalized by polydopamine and gentamicin.^68^ The hydrodynamic size and zeta potential of BN, PBN, and GPBN were determined using DLS (Table S1). The hydrodynamic diameter of BN was measured to be 176.5 nm. PBN slightly increased the hydrodynamic diameter to 196.3 nm. The nanoparticles exhibited a thin film of PDA surrounding them, which corresponded to a slight increase in the hydrodynamic diameter upon modification.^69^ The increase in size of the GPBN nanoconjugates (220.8 nm) was attributed to the attachment of the gentamicin on the surface of PBN.^61^ This could also be taken as evidence that the BN nanoparticles were successfully functionalized. Zeta potential values of BN, PBN, and GPBN are provided in Table S1. The pristine BN nanoparticles exhibited a negative potential value, −36.6 mV, due to the presence of B–OH and N–OH groups in the deionized water, which is consistent with the previous reported values for BN materials.^70^ Zeta potentials of PBN (−19.8 mV) became less negative upon PDA modification, which can be attributed to the loss of −OH groups as coated with polydopamine.^71,72^ Nevertheless, the GPBN particles exhibited a net charge of +6.2 mV, which was attributed to the presence of amine functionalities from the gentamicin on the surface of the nanoconjugates. This shift in zeta potential values from negative to positive provided confirmation of the attachment of gentamicin on the PBN nanoparticle surface through both Schiff base and Michael reactions.^61,62^ These results were consistent with previous findings, further validating the observations. To determine the crystalline features of the BN samples, XRD patterns were recorded for BN, PBN and GPBN, as shown in Figure 2. In the pattern of BN, the intensive characteristic peaks at 2θ = 26.9, 41.6, 43.9, 50.2, and 55.1° could be attributed to the (002), (100), (101), (102), and (004) crystallographic planes of hexagonal BN.^34,39^ After being modified with PDA and gentamicin , the pattern of PBN and GPBN was consistent with that of BN, indicating that their crystal structures were not damaged. In addition, no diffraction peaks have been observed for PDA due to its amorphous nature. To confirm the chemical constituents of all BN nanoconjugates, Raman and FTIR spectra were recorded. Figure S2 shows the Raman spectra of BN, PBN, and GPBN. The peak at 1366 cm^–1^ in the BN sample is attributed to the E_2g_ vibrational mode of BN.^43,72^ The Raman spectra of PBN and GPBN samples have a distinct peak at 1584 cm^–1^, which are attributed to the catechol deformation and stretching vibrations in the PDA structure.^73^ The FTIR spectra of BN, PBN and GPBN are shown in Figure S3. The bands at 1348 and 773 cm^–1^ in BN, PBN and GPBN samples are, respectively, assigned to the in-plane B–N stretching vibration and out-of-plane bending vibration.^68,74^ Compared with BN, PBN and GPBN show stronger peaks at around 2985 and 2902 cm^–1^, resulting from the methylene stretch vibration in PDA. The characteristic bands of PDA at 1498 and 1434 cm^–1^ corresponded to the stretching vibration of aromatic C=C bonds, while the bands in the range of 1400 to 1200 cm^–1^ were attributed to the bending vibration of −CH_2_ groups. The C–O asymmetric stretching vibration was not identified due to the overlap with a strong and broad peak at 1367 cm^–1^.^75^ As to PBN and GPBN, the bands assigned to the in-plane B–N stretching vibration became narrow and a weak band appeared at 1645 cm^–1^ corresponding to N–H bending vibration of secondary amino groups of PDA, which proved that PDA was successfully formed on BN nanoparticles.^75^ It is worth noting that gentamicin does not have a distinct feature in the IR spectrum. PDA and aminoglycosides have similar functionalities. The characteristic peaks of aminoglycoside antibiotics, such as the N–H bending frequency at around 1500 cm^–1^, overlap with the N–H amide peak of PDA. Also, due to the strong background coverage of BN and PDA, it is difficult to observe a clear gentamicin signal on the IR spectra.^61^

**Figure 3:**
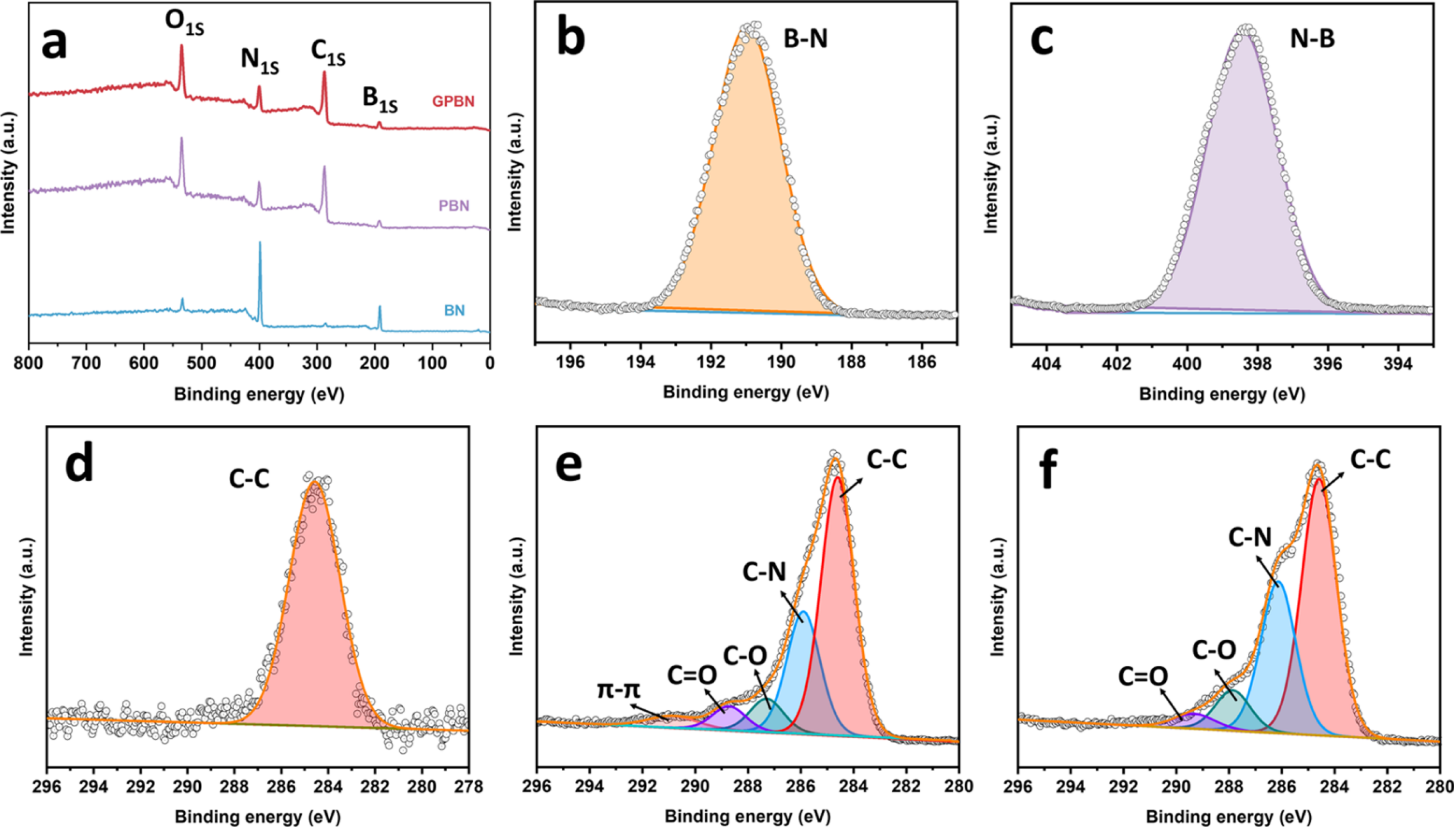
Full scan XPS spectra of BN, PBN, and GPBN (a). High-resolution B 1s (b) and N 1s (c) spectra of BN. High-resolution C 1s spectra of BN (d), PBN (e), and GPBN (f).

To gain a deeper understanding of the chemical characteristics of the BN nanoconjugate, its elemental composition and chemical state of the nanoconjugates were investigated by XPS. Figure 3a shows full XPS spectra of BN, PBN, and GPBN. All the samples showed peaks of B 1s, C 1s, O 1s, and N 1s in the full XPS spectra. According to the detection depth of XPS, the thickness of the PDA and gentamicin film on the BN surface was estimated to be less than 10 nm, which was consistent with previous reports.^55,57^ The atomic percentages belonging to each of the coating are shown in Table S2. The high-resolution B 1s and N 1s XPS spectra for BN is shown in Figure 3b,c with a single BE peak at 190.8 and 398.4 eV, respectively.^44^Figure 3d shows the high-resolution C 1s peak for BN with a single BE peak at 284.6 eV, which results from the contaminants. Compared with BN, the peak intensity of C 1s and O 1s increased while the B 1s and N 1s decreased after the functionalization with PDA. Besides, PBN shows an obvious increase in the N/B atomic ratio from 0.99 to 1.5, indicating the presence of a PDA layer on BN. The C 1s spectra were deconvoluted into distinct components exhibiting binding energies at 284.6, 285.9, 287.3, 288.7, and 290.9 eV which are assigned to C–C, C–N, C–O, C=O, and π–π bonds, respectively (Figure 3e).^76,77^ The element composition and chemical state of the XPS spectrum revealed the successful formation of PDA on BN nanoparticles. As to the GPBN nanoconjugate, the C 1s spectra could be deconvoluted into different components at 284.6, 286.1, 287.7, and 288.9 eV, which correspond to the aromatic C–C, C–N, C–O, and C=O bonds, respectively (Figure 3f). Moreover, it is found that the ratio corresponding to the relative intensities’ ratio of C–N and C–C increases from 0.47 to 0.59%, while the ratio corresponding to the relative intensities’ ratio of C–N and C–O increases from 2.13 to 2.66%. This is mainly attributed to the gentamicin loading.^62^

### 3.2 Gentamicin Loading and Release

The results of gentamicin loading to GPBN are presented in Figure S4a. It showed that the initial concentration of gentamicin from 50 to 500 μg/mL resulted in different amounts of loading to GPBN. An increase in the initial concentration of gentamicin resulted in a higher loading capacity relative to PBN. Increasing the concentration of gentamicin from 50 to 200 μg/mL resulted in an increase in the loading of gentamicin in PBN from 19.3 to 34.2 μg/mg. However, when the initial concentration of gentamicin was increased to 500 μg/mL (Figure S4a), the loading amount did not increase significantly. The release of gentamicin from GPBN was also investigated, as shown in Figure S4b. It showed that the increase in cumulative release between 24 and 48 h was relatively small compared to release over the first 24 h, indicating near-complete release of gentamicin in around 2 days. 33% of total drug was released to PBS from GPBN (which corresponds to 11.3 μg gentamicin released by 1 mg of GPBN). Some residual drug (67%) remained in GPBN. The release profile is similar to the previous report where PDA-coated curdlan hydrogel tested as the drug carrier.^59^ Most drugs are loaded on nanocarriers through non-covalent interactions or covalent bonds.^19^ In non-covalent interaction strategies, weak interactions between drugs and nanocarriers often lead to premature drug release. The covalent bond conjugation strategy can partially solve the premature and burst drug release problem. Gentamicin was loaded in PDA-modified BN nanoparticles in a relatively stable covalent manner. It is possible that the remaining drug may protect the GPBN nanoconjugate matrix against bacteria upon direct contact. Therefore, it is possible that gentamicin- and PDA-modified BN nanoparticles can still prevent bacterial infection after a certain degree of drug release. It was previously reported that about 81% of non-covalently interacting antibiotics could be released on the surface of BN nanoparticles.^46^ Therefore, it can be expected that by studying the interaction form of antibiotics and BN, different release modes can be obtained to achieve drug burst release or the possibility of providing long-term protection of nanocarriers.

### 3.3 GPBN Nanoconjugates Prevent Planktonic Bacterial Growth

**Figure 4:**
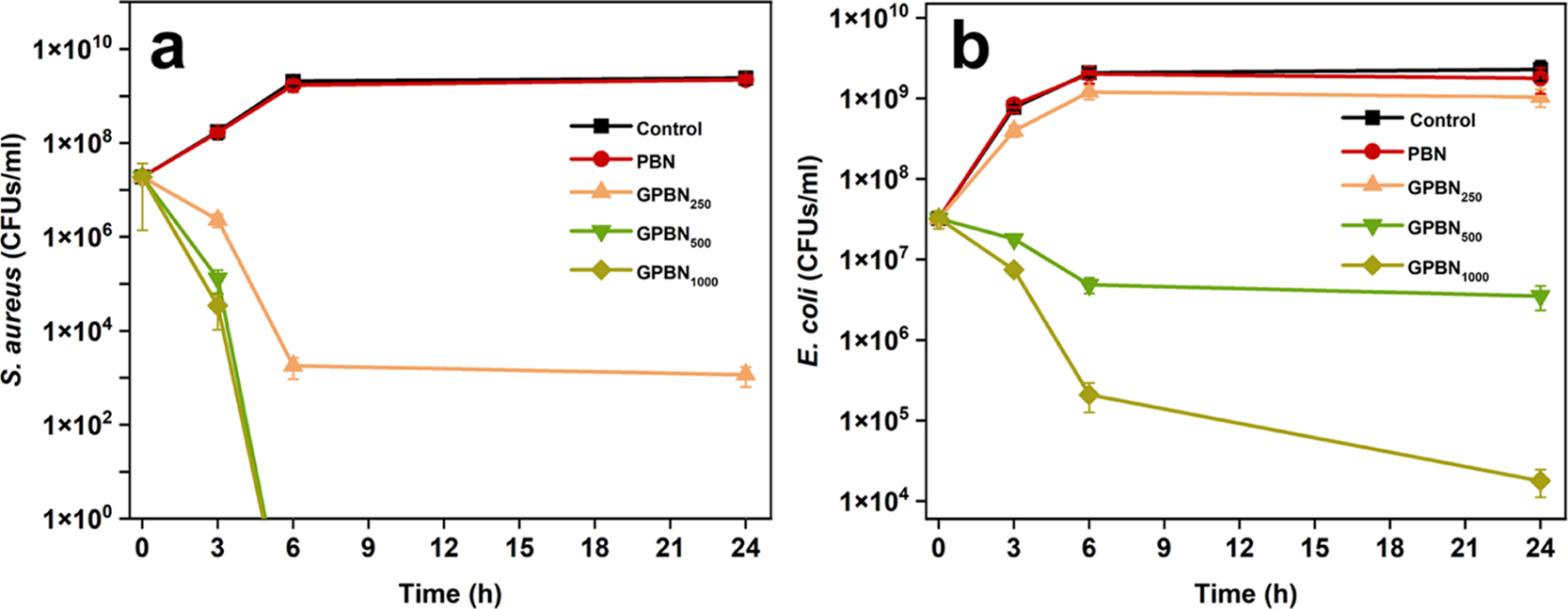
Time-kill studies of gentamicin of GPBN against *S. aureus* (a) and *E. coli* (b). The control represents the bacterial growth in culture medium without nanoconjugates. Data are presented as mean ± standard deviation from three independent biological replicates.

The antibacterial activity of GPBN nanoconjugates against the opportunistic pathogens *S. aureus* and *E. coli* was tested, as shown in Figure 4. BN nanoparticles without gentamicin and PDA loading did not demonstrate any antibacterial activity against both *S. aureus* and *E. coli* planktonic cells. This is consistent with previous reports that the pure BN nanoparticles have no noticeable effect against bacteria.^45,46^ Consistent with previous studies of nanoconjugates loaded with gentamycin, the effect of GPBN against *S. aureus* appeared to be time- and concentration-dependent.^61,78^ All tested concentrations were observed to be bactericidal against *S. aureus*, and their bactericidal efficiency was observed to increase over exposure time (Figure 4a). In addition, a concentration-dependent trend was observed with respect to the suppression of *S. aureus* growth. At higher concentrations (≥500 μg/mL) of GPBN, a significant reduction in cfu count was observed after 3 h, and no surviving cells were detected after 6 h of exposure. At a lower concentration (250 μg/mL), GPBN showed the activity against *S. aureus* causing a reduction by a factor of 1 000 000 (6 log_10_ units) in bacterial viability within 24 h. However, *E. coli* was observed to be less sensitive than *S. aureus* (Figure 4b). When GPBN was tested on *E. coli*, no significant effect was observed at 250 μg/mL. The *E. coli* strain (UTI89) used in this study is comparatively resistant (in compared to *S. aureus*) to gentamycin. That is the main reason for the difference in antimicrobial activity. GPBN at concentrations of 500 and 1000 μg/mL reduced the viability of *E. coli* by a factor of 100 (2 log_10_ units) and 100 000 (5 log_10_ units), respectively, after 24 h of treatment. Although differences were observed in the antibacterial effects of GPBN on *S. aureus* and *E. coli*, it was concluded that GPBN effectively prevents planktonic growth and caused a time- and concentration-dependent killing against both species.

### 3.4 GPBN Nanoconjugates Successfully Kill Bacterial Biofilms

**Figure 5:**
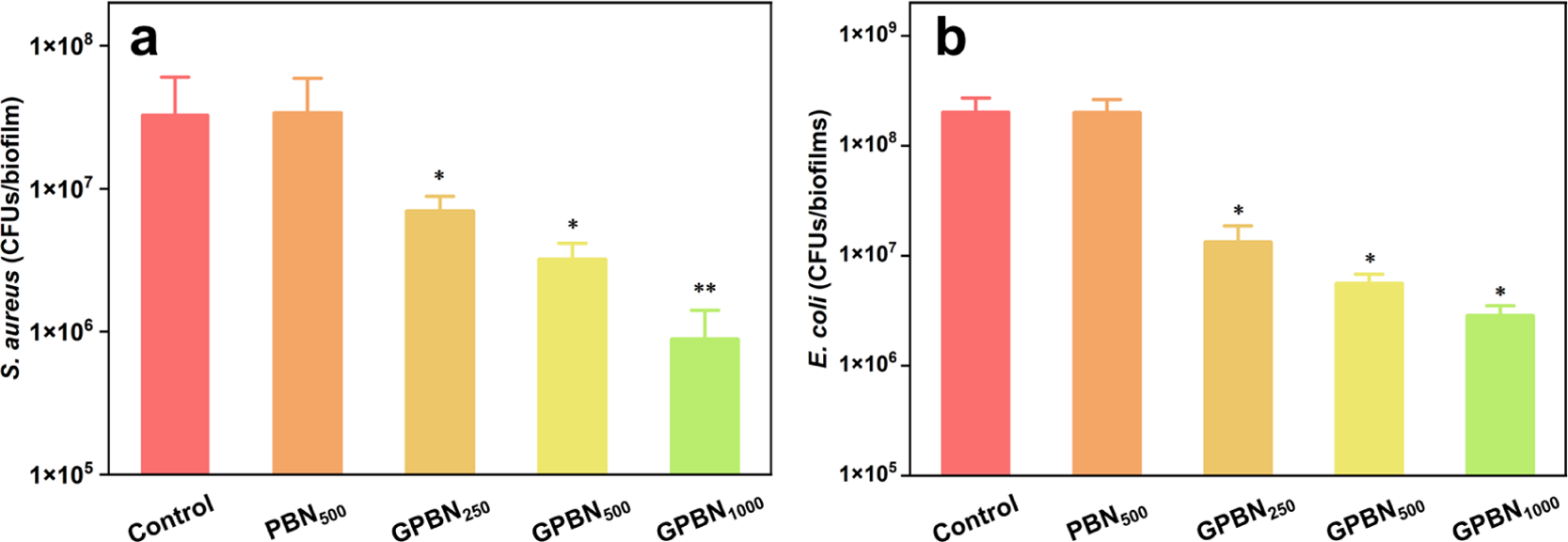
Effect of GPBN against bacterial biofilms. cfu count after treatment with different concentrations of GPBN against (a) *S. aureus* and (b) *E. coli*. The control represents the culture medium without nanoconjugates. Data represent mean ± standard deviation error. *p < 0.05; **p < 0.01.

**Figure 6:**
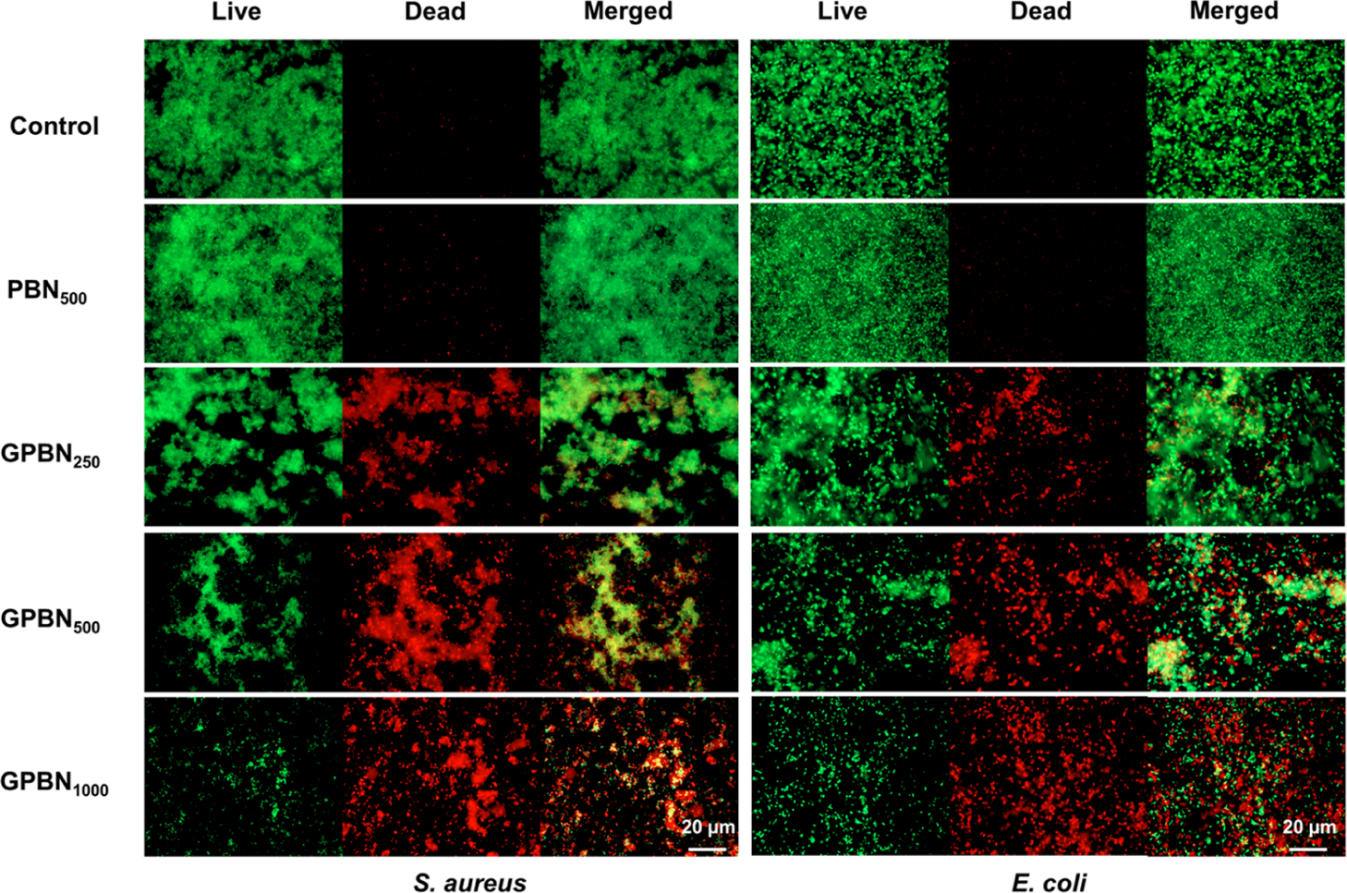
Live/dead staining images of preformed *S. aureus* and *E. coli* biofilms treated with different concentrations of GPBN and observed by fluorescence microscopy. The control represents the culture medium without nanoconjugates.

**Figure 7:**
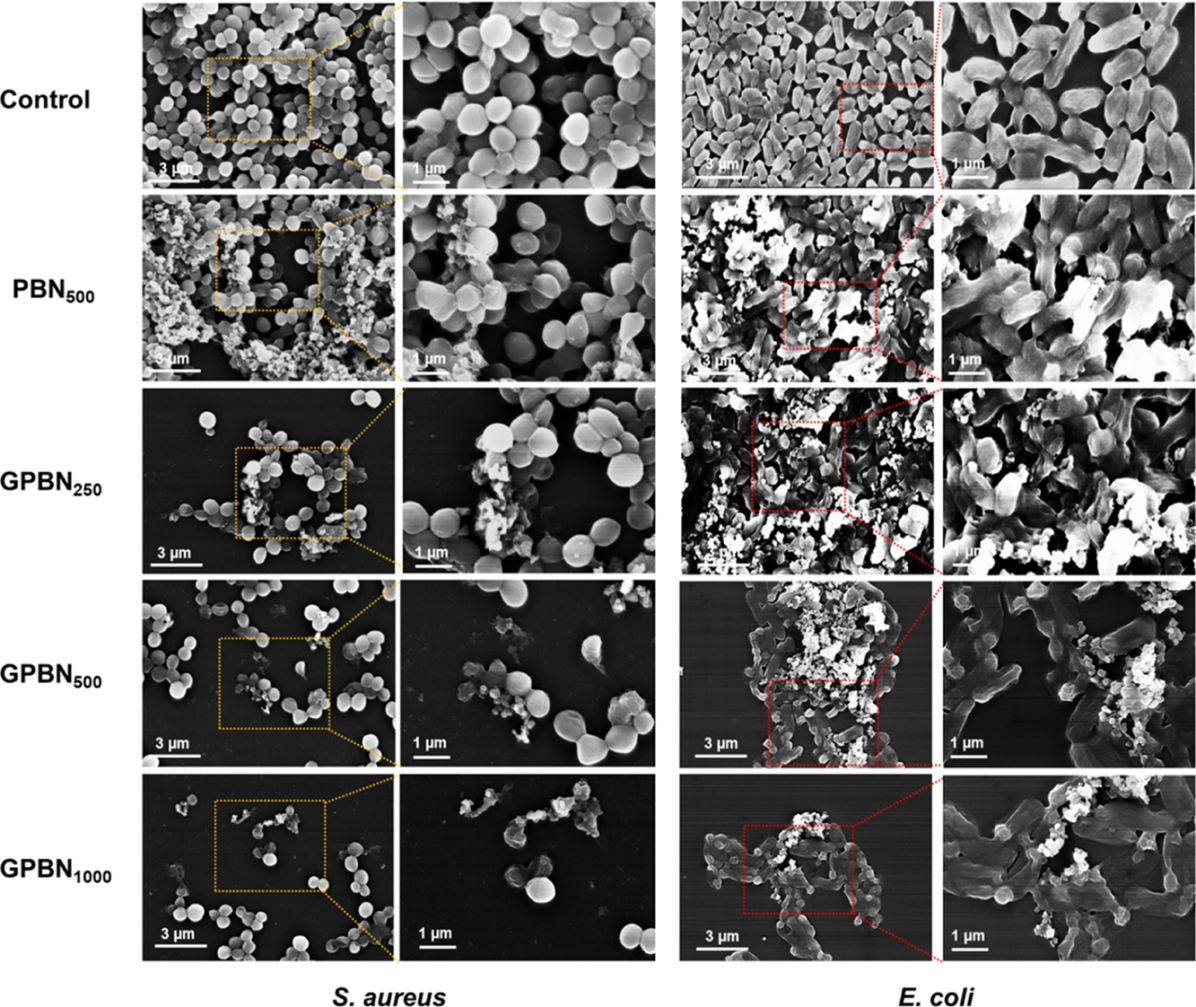
SEM images of *S. aureus* and *E. coli* biofilms treated with different concentrations of GPBN. The control represents the culture medium without nanoconjugates.

Biofilms have a major impact in the field of medical devices and biomedicine.^1,79^ The potential of GPBN nanoconjugates against pre-formed biofilms of *S. aureus* and *E. coli* was assessed. As shown in Figure 5, PBN without gentamicin did not demonstrate any antibiofilm activity against both *S. aureus* and *E. coli* biofilms. This was in line with previous reports on BN and polydopamine nanomaterials.^45,80^ In contrast, the mature biofilms treated with GPBN were disrupted and the viability of bacterial cells in biofilms was significantly reduced by >90% in both *S. aureus* and *E. coli* biofilms. Additionally, live cells (stained in green) and dead cells (stained in red) on the surface of the biofilm were visualized under a fluorescence microscope using live/dead staining (Figure 6). Compared with the control sample, negligible cell death and no reduction in bacterial adhesion were observed after PBN exposure. However, *S. aureus* and *E. coli* biofilms showed strong red fluorescence signals compared to the control group after the treatment with GPBN, indicating that the bacterial cells in biofilms were killed. The area ratio of dead cells and living cells was used to show the degree of biofilm damage after the exposure of GPBN nanoconjugates. Live/dead staining images of biofilms were analyzed by ImageJ, as shown in Figure S5. Compared with the control samples, the area ratios of dead cells and live cells hardly changed after PBN exposure and both tended to zero. However, the area ratios were greatly increased after treatment with GPBN, indicating that the bacterial cells in the biofilms were killed. Moreover, the area ratios of dead cells and live cells were found to be concentration-dependent with GPBN treatment. In addition, the treated biofilms were examined using SEM. Biofilm morphology was significantly altered after GPBN treatment (Figure 7). The severity of biofilm disruption correlated with the concentration of GPBN treatment. GPBN nanoconjugates might be adsorbed and adhered to the biofilm surface. Besides, GPBN penetrates the biofilm matrix to disturb the bacterial communities, where it enhances the cell wall damage by increasing the permeability of cell membranes, leading to deactivation of bacteria within the biofilm. Meanwhile, gentamicin could enter the bacterial cell and kill the bacteria through the inhibition of protein synthesis by binding of gentamicin to the ribosomal subunits.^81,82^

### 3.5 GPBN Nanoconjugate Coating Effectively Prevents Biofilm Formation

**Figure 8:**
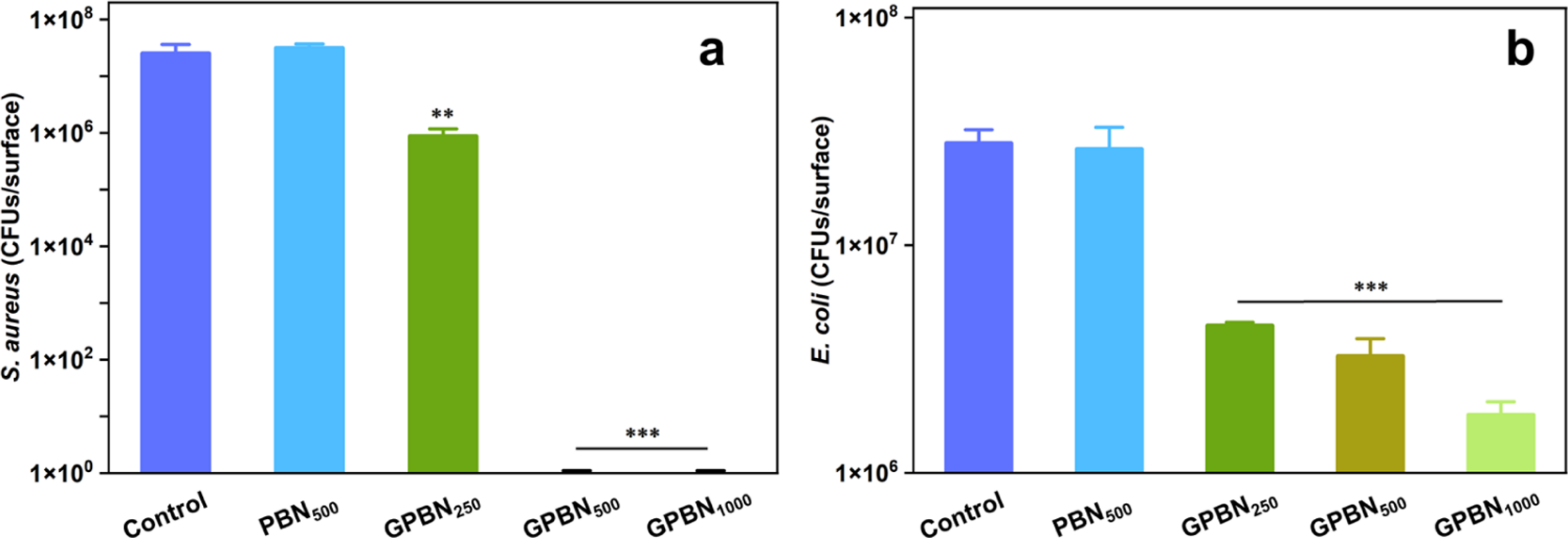
Measurement of biofilm inhibitory efficiency of GPBN coatings against (a) *S. aureus* and (b) *E. coli*. Bacterial viability, quantified as cfu counts, was evaluated following 24 h of growth on GPBN-coated and non-coated (control) surfaces. The data represent the mean ± standard deviation from three independent biological replicates. *p < 0.05; **p < 0.01; ***p < 0.001.

Preventing biofilm formation is even more valuable for protecting biomedical devices than killing biofilms.^5^ Here, we asked whether our GPBN nanoconjugates could be used as coatings to prevent *S. aureus* and *E. coli* from forming biofilms on protected surfaces. The morphology of the GPBN coatings ire shown in Figure S6. cfu counts were used to assess the viability of bacteria in the formed biofilm. Coatings of PBN showed no antimicrobial effect. However, coatings based on GPBN inhibited *S. aureus* biofilm formation (Figure 8a), with no cell attachment on surfaces protected with the ≥500 μg/mL GPBN. The preventive effect of the coatings against *E. coli* biofilms was considerably weaker, only about 10-fold with the highest GPBN concentration (Figure 8b). This difference may be due to the lower sensitivity of the *E. coli* strain to gentamicin compared to the *S. aureus* strain used in this work.

**Figure 9:**
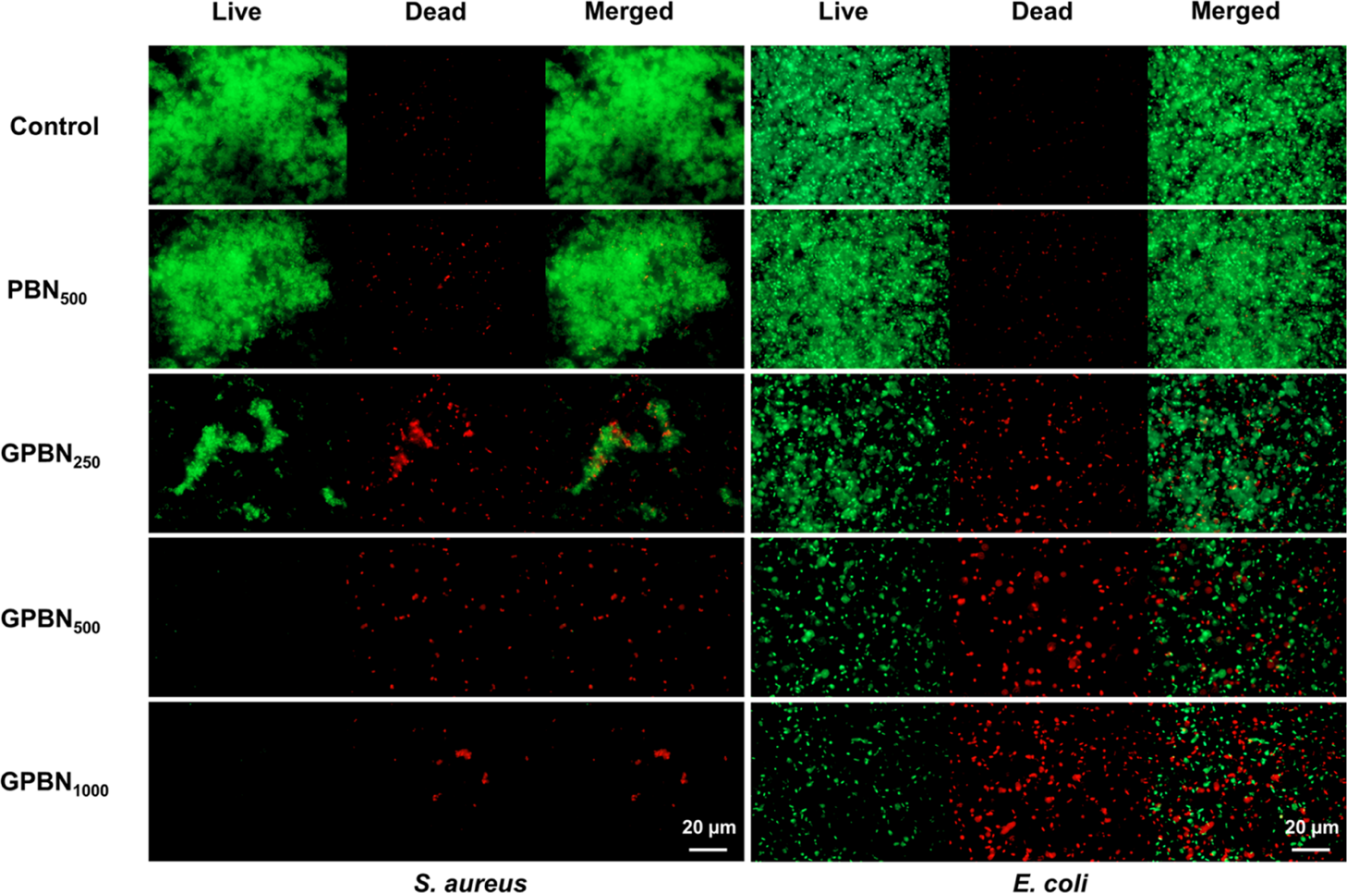
Live/dead staining images of *S. aureus* and *E. coli* biofilms was performed after 24 h of growth on GPBN-coated and non-coated (control) surfaces.

The reduction in cfu counts could be attributed to the coating’s resistance to bacterial adhesion or the bactericidal activity resulting from the release of gentamicin from the coating. In order to differentiate between these two effects and validate the findings derived from cfu counting, biofilms were stained with live/dead fluorescence probes and visualized under a fluorescence microscope (Figure 9). If the coating is bactericidal, dead cells (stained red) will be observed on the surface. The strong bactericidal activity may also affect the early colonization of bacteria due to the release of drug in the medium. If the coating prevents bacterial adhesion, a smaller number of bacteria can be observed on the coated surface compared to the non-coated counterparts. It was observed that almost no dead cells were observed on the control coating, and little loss of bacterial adhesion was observed with PBN coatings, demonstrating that PBN cannot kill the bacteria. However, there was an appearance of dead cells (stained red) on the GPBN coatings, along with a reduced number of attached bacterial cells. These observations indicate that the GPBN coatings not only possess bactericidal properties but also effectively hinder the initial stages of bacterial surface colonization.

To further examine the biocompatibility of BN nanoconjugates, the Huh 7 human liver cell line was used for this study. As shown in Figure S7, there was almost no decrease in cell viability when the concentration of BN nanoconjugates neared 1000 μg/mL, which indicates that the as-synthesized BN nanoconjugates possessed excellent biocompatibility. From the above results, GPBN exhibited effective antibacterial effect. As previously discussed in the literature, the potential mechanism underlying the antibacterial effect of GPBN nanoparticles can be attributed to the interaction between the positively charged nanoconjugates’ surface and the bacterial membrane.^83,84^ The net positive charge of GPBN favors electrostatic interactions with the negatively charged bacterial cell surface. Post interaction, the GPBN nanoparticles can impede the entry of crucial nutrients and the release of metabolic waste products, thereby inhibiting bacterial growth. In addition, cationic charged nanoparticles have been reported to have the ability to disrupt microbial cell membranes.^61^ As shown in Figure 7, SEM images showed extensive damage to the cell surface of the bacteria, which would lead to the release of the intracellular components. These changes in morphological features suggest that GPBN irreversibly damages the bacterial cell when it interacts with the cell. Fluorescence microscopy images after staining with SYTO9 and potassium iodide (PI) can provide insights into the impact of GPBN on cell membrane integrity. Membrane-permeable dye SYTO9 can penetrate live bacteria for staining. However, membrane-impermeable PI can only penetrate the damaged membrane of dead cells. When the cell membrane is damaged, due to the permeable membrane, PI can enter the cell and bind to DNA. Consequently, dead bacteria exhibit intense red fluorescence due to the binding of PI.^85^ As depicted in Figure 6, the control *S. aureus* and *E. coli* cells exhibited green staining, signifying their viability and live state. In contrast, cells treated with GPBN exhibited red fluorescence. This observation confirms that GPBN compromised cell membrane integrity and outer membrane permeability, ultimately resulting in cell death. In addition, during the interaction of the GPBN with the bacterial cell membrane, the nanoconjugates can release gentamicin. Aminoglycosides have the ability to penetrate the cell membrane. Once inside the cell, they bind to the ribosomal 30S receptor unit, disrupting RNA synthesis and causing a blockade in protein synthesis that can lead to bacterial death.^82^ The next step will focus on verifying the mechanism of action of the GPBN on cells and biofilms, such as oxidative stress, proteomics, etc.^86,87^

The BN nanoconjugates in this work can be expected not only as an antimicrobial coating grafted onto the surface of in vivo implants to prevent biofilm infection but also as a nano-drug delivery system to treat biofilms. The current research on the in vivo toxicology of BN nanomaterials is still in its early stages. Studies involving BN nanomaterials, whether pristine or functionalized, have been carried out by administering them to animals via injection or ingestion. Previous studies have reported that BN is biocompatible at specific doses and exposure times when used in vivo.^88^ However, Xin et al. revealed that high doses of BN nanotubes led to acute lung inflammation and injury after 7 days of exposure to lung cells.^89^ Similarly, Kodali et al. observed that the commercial grade BN nanotubes resulted in dose-dependent and acute inflammation in vivo, leading to increased levels of lactic dehydrogenase activity in bronchoalveolar lavage fluid, polymorphonuclear cell influx in the lungs, loss of mitochondrial membrane potential, and increased accumulation of 4-hydroxynonenal.^90^ Another limitation of BN is its lack of biodegradability. Soares et al. demonstrated that glycol chitosan-functionalized BN nanotubes were injected into the tails of Swiss mice to assess their biodistribution. The results showed that after 24 h, BN nanotubes had accumulated in the liver, spleen, and intestine.^91^ Furthermore, the use of BN in vivo is limited by our insufficient understanding of its long-term effects on living organisms. Although some research has been conducted on the biocompatibility and toxicity of BN, further investigation is necessary to fully comprehend the long-term consequences of this material on living organisms.^28^

## 4 Conclusions

This study reports an environmentally friendly and novel nanomaterial composite based on BN, with pronounced antibacterial and biofilm inhibitory properties. The formation of the GPBN conjugate was confirmed by SEM, TEM, XPS, FTIR, and Raman analysis. GPBN exhibited excellent antibacterial and antibiofilm activities against *S. aureus* and *E. coli*. Cell viability was significantly reduced in planktonic cells and biofilms exposed to GPBN. Fluorescence microscopy and SEM images clearly demonstrate the deactivation of biofilm cells, and this is consistent with the viability results of gentamicin-assembled nanoconjugates. In addition, we propose that GPBN could constitute a protective surface coating against biofilm formation. Translating the findings of in vitro studies to the real-world scenario of biofilm infection in vivo can be challenging. In vitro studies often involve loading large quantities of bacteria onto surfaces, creating a more conducive environment for biofilm formation compared to the in vivo conditions. Although it is not possible to directly compare the in vitro and in vivo situations, we believe that the obtained results hold significant importance for the continued advancement and development of BN-based nanocarriers, and this BN nanoconjugate could be a promising method of preventing biofilm-related infections.

## Supporting Information Available

SEM image of BN, PBN and GPBN and EDS elemental mapping of GPBN; hydrodynamic size and zeta potential of the BN, PBN and GPBN; surface elemental composition of BN, PBN and GPBN; Raman spectra of BN, PB, and GPBN; FTIR spectra of BN, PBN, and GPBN; amount of gentamicin loading and release for GPBN; area ratio of dead/live cells of *S. aureus* and *E. coli* biofilms after exposure of GPBN; SEM images of GPBN coatings; AlamarBlue cell viability 24 h after administration of BN, PBN, and GPBN (PDF)

## References

[ref1] Percival S. L.; Suleman L.; Vuotto C.; Donelli G. Healthcare-associated Infections, Medical Devices and Biofilms: Risk, Tolerance and Control. J. Med. Microbiol. 2015, 64, 323–334. 10.1099/jmm.0.000032. 25670813

[ref2] Stewart P. S.; William Costerton J. Antibiotic Resistance of Bacteria in Biofilms. Lancet 2001, 358, 135–138. 10.1016/s0140-6736(01)05321-1. 11463434

[ref3] Singh A.; Amod A.; Pandey P.; Bose P.; Pingali M. S.; Shivalkar S.; Varadwaj P. K.; Sahoo A. K.; Samanta S. K. Bacterial Biofilm Infections, Their Resistance to Antibiotics Therapy and Current Treatment Strategies. Biomed. Mater. 2022, 17, 022003 10.1088/1748-605x/ac50f6. 35105823

[ref4] Xin Q.; Shah H.; Nawaz A.; Xie W.; Akram M. Z.; Batool A.; Tian L.; Jan S. U.; Boddula R.; Guo B.; Liu Q.; et al. Antibacterial Carbon-based Nanomaterials. Adv. Mater. 2019, 31, 1804838 10.1002/adma.201804838. 30379355

[ref5] Liu Y.; Shi L.; Su L.; van der Mei H. C.; Jutte P. C.; Ren Y.; Busscher H. J. Nanotechnology-based Antimicrobials and Delivery Systems for Biofilm-infection Control. Chem. Soc. Rev. 2019, 48, 428–446. 10.1039/c7cs00807d. 30601473

[ref6] Xiu W.; Shan J.; Yang K.; Xiao H.; Yuwen L.; Wang L. Recent development of nanomedicine for the treatment of bacterial biofilm infections. View 2021, 2, 20200065 10.1002/viw.20200065.

[ref7] Wang Y.; Zhou J.; Yuan L.; Wu F.; Xie L.; Yan X.; Li H.; Li Y.; Shi L.; Hu R.; Liu Y. Neighboring Carboxylic Acid Boosts Peroxidase - Like Property of Metal - Phenolic Nano -Networks in Eradicating Streptococcus mutans Biofilms. Small 2023, 19, 2206657 10.1002/smll.202206657. 36394193

[ref8] Wu F.; Ma J.; Wang Y.; Xie L.; Yan X.; Shi L.; Li Y.; Liu Y. Single copper atom photocatalyst powers an integrated catalytic cascade for drug-resistant bacteria elimination. ACS Nano 2023, 17, 2980–2991. 10.1021/acsnano.2c11550. 36695402

[ref9] Zhang J.; Bai Q.; Bi X.; Zhang C.; Shi M.; Yu W. W.; Du F.; Wang L.; Wang Z.; Zhu Z.; Sui N. Piezoelectric enhanced peroxidase-like activity of metal-free sulfur doped graphdiyne nanosheets for efficient water pollutant degradation and bacterial disinfection. Nano Today 2022, 43, 101429 10.1016/j.nantod.2022.101429.

[ref10] Li H.; Gong M.; Xiao J.; Hai L.; Luo Y.; He L.; Wang Z.; Deng L.; He D. Photothermally activated multifunctional MoS_2_ bactericidal nanoplatform for combined chemo/ photothermal/photodynamic triple-mode therapy of bacterial and biofilm infections. Chem. Eng. J. 2022, 429, 132600 10.1016/j.cej.2021.132600.

[ref11] Xue K.; Yang C.; Wang C.; Liu Y.; Liu J.; Shi L.; Zhu C. An Exceptional Broad-Spectrum Nanobiocide for Multimodal and Synergistic Inactivation of Drug-Resistant Bacteria. CCS Chem. 2022, 4, 272–285. 10.31635/ccschem.021.202000714.

[ref12] Gupta A.; Mumtaz S.; Li C. H.; Hussain I.; Rotello V. M. Combatting antibiotic-resistant bacteria using nanomaterials. Chem. Soc. Rev. 2019, 48, 415–427. 10.1039/c7cs00748e. 30462112 PMC6340759

[ref13] Liu J.; Cui L.; Losic D. Graphene and graphene oxide as new nanocarriers for drug delivery applications. Acta Biomater. 2013, 9, 9243–9257. 10.1016/j.actbio.2013.08.016. 23958782

[ref14] Sontakke A. D.; Tiwari S.; Purkait M. K. A comprehensive review on graphene oxide-based nanocarriers: Synthesis, functionalization and biomedical applications. FlatChem 2023, 38, 100484 10.1016/j.flatc.2023.100484.

[ref15] Ayaz M.; Ullah F.; Sadiq A.; Ullah F.; Ovais M.; Ahmed J.; Devkota H. P. Synergistic interactions of phytochemicals with antimicrobial agents: Potential strategy to counteract drug resistance. Chem.-Biol. Interact. 2019, 308, 294–303. 10.1016/j.cbi.2019.05.050. 31158333

[ref16] Huang F.; Cai X.; Hou X.; Zhang Y.; Liu J.; Yang L.; Liu Y.; Liu J. A dynamic covalent polymeric antimicrobial for conquering drug - resistant bacterial infection. Exploration 2022, 2, 20210145 10.1002/exp.20210145. 37325499 PMC10191036

[ref17] Pushpalatha R.; Selvamuthukumar S.; Kilimozhi D. Nanocarrier mediated combination drug delivery for chemotherapy-A review. J. Drug Delivery Sci. Technol. 2017, 39, 362–371. 10.1016/j.jddst.2017.04.019.

[ref18] Di Y.; Gao Y.; Gai X.; Wang D.; Wang Y.; Yang X.; Zhang D.; Pan W.; Yang X. Co-delivery of hydrophilic gemcitabine and hydrophobic paclitaxel into novel polymeric micelles for cancer treatment. RSC Adv. 2017, 7, 24030–24039. 10.1039/c7ra02909h.

[ref19] Ding Y.; Hu X.; Piao Y.; Huang R.; Xie L.; Yan X.; Sun H.; Li Y.; Shi L.; Liu Y. Lipid Prodrug Nanoassemblies via Dynamic Covalent Boronates. ACS Nano 2023, 17, 6601–6614. 10.1021/acsnano.2c12233. 36999933

[ref20] Zhan Y.; Hu X.; Li Y.; Wang Y.; Chen H.; Omolo C. A.; Govender T.; Li H.; Huang F.; Shi L.; Hu X. Antimicrobial hybrid amphiphile via dynamic covalent bonds enables bacterial biofilm dispersal and bacteria eradication. Adv. Funct. Mater. 2023, 33, 2214299 10.1002/adfm.202214299.

[ref21] Li Y.; Piao Y. Z.; Chen H.; Shi K.; Dai J.; Wang S.; Zhou T.; Le A. T.; Wang Y.; Wu F.; Ma R.; et al. Dynamic covalent nano-networks comprising antibiotics and polyphenols orchestrate bacterial drug resistance reversal and inflammation alleviation. Bioact. Mater. 2023, 27, 288–302. 10.1016/j.bioactmat.2023.04.014. 37113688 PMC10126917

[ref22] Wang J.; Ma F.; Sun M. Graphene, Hexagonal Boron Nitride, and Their Heterostructures: Properties and Applications. RSC Adv. 2017, 7, 16801–16822. 10.1039/c7ra00260b.

[ref23] Zhang K.; Feng Y.; Wang F.; Yang Z.; Wang J. Two Dimensional Hexagonal Boron Nitride (2D-hBN): Synthesis, Properties and Applications. J. Mater. Chem. C 2017, 5, 11992–12022. 10.1039/c7tc04300g.

[ref24] Weng Q.; Wang X.; Wang X.; Bando Y.; Golberg D. Functionalized Hexagonal Boron Nitride Nanomaterials: Emerging Properties and Applications. Chem. Soc. Rev. 2016, 45, 3989–4012. 10.1039/c5cs00869g. 27173728

[ref25] Roy S.; Zhang X.; Puthirath A. B.; Meiyazhagan A.; Bhattacharyya S.; Rahman M. M.; Babu G.; Susarla S.; Saju S. K.; Tran M. K.; Sassi L. M.; et al. Structure, Properties and Applications of Two-dimensional Hexagonal Boron Nitride. Adv. Mater. 2021, 33, 2101589 10.1002/adma.202101589. 34561916

[ref26] Bao J.; Jeppson K.; Edwards M.; Fu Y.; Ye L.; Lu X.; Liu J. Synthesis and Applications of Two-dimensional Hexagonal Boron Nitride in Electronics Manufacturing. Electron. Mater. Lett. 2016, 12, 1–16. 10.1007/s13391-015-5308-2.

[ref27] Pan Y.; Zheng H.; Li G.; Li Y.; Jiang J.; Chen J.; Xie Q.; Wu D.; Ma R.; Liu X.; Xu S.; et al. Antibiotic-Like Activity of Atomic Layer Boron Nitride for Combating Resistant Bacteria. ACS Nano 2022, 16, 7674–7688. 10.1021/acsnano.1c11353. 35511445

[ref28] Merlo A.; Mokkapati V. R. S. S.; Pandit S.; Mijakovic I. Boron Nitride Nanomaterials: Biocompatibility and Bio-applications. Biomater. Sci. 2018, 6, 2298–2311. 10.1039/c8bm00516h. 30059084

[ref29] Hilder T. A.; Gaston N. Interaction of Boron Nitride Nanosheets with Model Cell Membranes. ChemPhysChem 2016, 17, 1573–1578. 10.1002/cphc.201600165. 26934705

[ref30] Pandit S.; Gaska K.; Mokkapati V. R. S. S.; Forsberg S.; Svensson M.; Kádár R.; Mijakovic I. Antibacterial Effect of Boron Nitride Flakes with Controlled Orientation in Polymer Composites. RSC Adv. 2019, 9, 33454–33459. 10.1039/c9ra06773f. 35529107 PMC9073355

[ref31] Xiong S. W.; Fu P. G.; Zou Q.; Chen L. Y.; Jiang M. Y.; Zhang P.; Wang Z. G.; Cui L. S.; Guo H.; Gai J. G. Heat Conduction and Antibacterial Hexagonal Boron Nitride/Polypropylene Nanocomposite Fibrous Membranes for Face Masks with Long-time Wearing Performance. ACS Appl. Mater. Interfaces 2020, 13, 196–206. 10.1021/acsami.0c17800. 33356094

[ref32] Zhi C.; Bando Y.; Tang C.; Honda S.; Sato K.; Kuwahara H.; Golberg D. Covalent Functionalization: towards Soluble Multiwalled Boron Nitride Nanotubes. Angew. Chem., Int. Ed. 2005, 44, 7932–7935. 10.1002/anie.200502846. 16287164

[ref33] Permyakova E. S.; Antipina L. Y.; Kovalskii A. M.; Zhitnyak I. Y.; Gudz K. Y.; Polčak J.; Sorokin P. B.; Manakhov A. M.; Shtansky D. V. Experimental and Theoretical Study of Doxorubicin Physicochemical Interaction with BN (O) Drug Delivery Nanocarriers. J. Phys. Chem. C 2018, 122, 26409–26418. 10.1021/acs.jpcc.8b07531.

[ref34] Hao L.; Gong L.; Chen L.; Guan M.; Zhou H.; Qiu S.; Wen H.; Chen H.; Zhou X.; Akbulut M. Composite Pesticide Nanocarriers Involving Functionalized Boron Nitride Nanoplatelets for pH-responsive Release and Enhanced UV Stability. Chem. Eng. J. 2020, 396, 125233 10.1016/j.cej.2020.125233.

[ref35] Sharker S. M. Hexagonal Boron Nitrides (White Graphene): A Promising Method for Cancer Drug Delivery. Int. J. Nanomed. 2019, 14, 9983–9993. 10.2147/ijn.s205095. PMC6927571 31908454

[ref36] Permyakova E. S.; Sukhorukova I. V.; Antipina L. Y.; Konopatsky A. S.; Kovalskii A. M.; Matveev A. T.; Lebedev O. I.; Golberg D. V.; Manakhov A. M.; Shtansky D. V. Synthesis and Characterization of Folate Conjugated Boron Nitride Nanocarriers for Targeted Drug Delivery. J. Phys. Chem. C 2017, 121, 28096–28105. 10.1021/acs.jpcc.7b10841.

[ref37] Weng Q.; Wang B.; Wang X.; Hanagata N.; Li X.; Liu D.; Wang X.; Jiang X.; Bando Y.; Golberg D. Highly Water-soluble, Porous, and Biocompatible Boron Nitrides for Anticancer Drug Delivery. ACS Nano 2014, 8, 6123–6130. 10.1021/nn5014808. 24797563

[ref38] Sukhorukova I. V.; Zhitnyak I. Y.; Kovalskii A. M.; Matveev A. T.; Lebedev O. I.; Li X.; Gloushankova N. A.; Golberg D.; Shtansky D. V. Boron Nitride Nanoparticles with A Petal-like Surface as Anticancer Drug-delivery Systems. ACS Appl. Mater. Interfaces 2015, 7, 17217–17225. 10.1021/acsami.5b04101. 26192448

[ref39] Kıvanç M.; Barutca B.; Koparal A. T.; Göncü Y.; Bostancı S. H.; Ay N. Effects of Hexagonal Boron Nitride Nanoparticles on Antimicrobial and Antibiofilm Activities, Cell Viability. Mater. Sci. Eng.,C 2018, 91, 115–124. 10.1016/j.msec.2018.05.028. 30033238

[ref40] Mukheem A.; Shahabuddin S.; Akbar N.; Miskon A.; Muhamad Sarih N.; Sudesh K.; Ahmed Khan N.; Saidur R.; Sridewi N. Boron Nitride Doped Polyhydroxyalkanoate/chitosan Nanocomposite for Antibacterial and Biological Applications. Nanomaterials 2019, 9, 645 10.3390/nano9040645. 31010071 PMC6523564

[ref41] Smith McWilliams A. D.; Martinez-Jimenez C.; Matatyaho Ya’akobi A.; Ginestra C. J.; Talmon Y.; Pasquali M.; Martí A. A. Understanding the Exfoliation and Dispersion of Hexagonal Boron Nitride Nanosheets by Surfactants: Implications for Antibacterial and Thermally Resistant Coatings. ACS Appl. Nano Mater. 2021, 4, 142–151. 10.1021/acsanm.0c02437.

[ref42] Nasr M.; Soussan L.; Viter R.; Eid C.; Habchi R.; Miele P.; Bechelany M. High Photodegradation and Antibacterial Activity of BN–Ag/TiO_2_ Composite Nanofibers under Visible Light. New J. Chem. 2018, 42, 1250–1259. 10.1039/c7nj03183a.

[ref43] Maria Nithya J. S.; Pandurangan A. Aqueous Dispersion of Polymer Coated Boron Nitride Nanotubes and Their Antibacterial and Cytotoxicity Studies. RSC Adv. 2014, 4, 32031–32046. 10.1039/c4ra04846f.

[ref44] Guan M.; Hao L.; Chen L.; Gao F.; Qiu S.; Zhou H.; Chen H.; Zhou X. Facile Mechanical-induced Functionalization of Hexagonal Boron Nitride and Its Application as Vehicles for Antibacterial Essential Oil. ACS Sustainable Chem. Eng. 2020, 8, 15120–15133. 10.1021/acssuschemeng.0c03781.

[ref45] Gudz K. Y.; Permyakova E. S.; Matveev A. T.; Bondarev A. V.; Manakhov A. M.; Sidorenko D. A.; Filippovich S. Y.; Brouchkov A. V.; Golberg D. V.; Ignatov S. G.; Shtansky D. V. Pristine and Antibiotic-loaded Nanosheets/Nanoneedles-based Boron Nitride Films as A Promising Platform to Suppress Bacterial and Fungal Infections. ACS Appl. Mater. Interfaces 2020, 12, 42485–42498. 10.1021/acsami.0c10169. 32845601

[ref46] Gudz K. Y.; Antipina L. Y.; Permyakova E. S.; Kovalskii A. M.; Konopatsky A. S.; Filippovich S. Y.; Dyatlov I. A.; Slukin P. V.; Ignatov S. G.; Shtansky D. V. Ag-doped and Antibiotic-loaded Hexagonal Boron Nitride Nanoparticles as Promising Carriers to Fight Different Pathogens. ACS Appl. Mater. Interfaces 2021, 13, 23452–23468. 10.1021/acsami.1c03775. 34000197

[ref47] Lee H.; Dellatore S. M.; Miller W. M.; Messersmith P. B. Mussel-inspired Surface Chemistry for Multifunctional Coatings. Science 2007, 318, 426–430. 10.1126/science.1147241. 17947576 PMC2601629

[ref48] Liu M.; Zeng G.; Wang K.; Wan Q.; Tao L.; Zhang X.; Wei Y. Recent Developments in Polydopamine: An Emerging Soft Matter for Surface Modification and Biomedical Applications. Nanoscale 2016, 8, 16819–16840. 10.1039/c5nr09078d. 27704068

[ref49] Li Y.; Li C.; Yu R.; Ding Y. Application of polydopamine on the implant surface modification. Polym. Bull. 2022, 79, 5613–5633. 10.1007/s00289-021-03793-9.

[ref50] Fu Y.; Yang L.; Zhang J.; Hu J.; Duan G.; Liu X.; Li Y.; Gu Z. Polydopamine antibacterial materials. Mater. Horiz. 2021, 8, 1618–1633. 10.1039/d0mh01985b. 34846495

[ref51] Awasthi A. K.; Gupta S.; Thakur J.; Gupta S.; Pal S.; Bajaj A.; Srivastava A. Polydopamine-on-liposomes: Stable nanoformulations, uniform coatings and superior antifouling performance. Nanoscale 2020, 12, 5021–5030. 10.1039/c9nr07770g. 32065189

[ref52] Alves D.; Vaz A. T.; Grainha T.; Rodrigues C. F.; Pereira M. O. Design of an antifungal surface embedding liposomal amphotericin B through a mussel adhesive-inspired coating strategy. Front. Chem. 2019, 7, 431 10.3389/fchem.2019.00431. 31275922 PMC6591271

[ref53] Xu X.; Wang L.; Luo Z.; Ni Y.; Sun H.; Gao X.; Li Y.; Zhang S.; Li Y.; Wei S. Facile and versatile strategy for construction of anti-inflammatory and antibacterial surfaces with polydopamine-mediated liposomes releasing dexamethasone and minocycline for potential implant applications. ACS Appl. Mater. Interfaces 2017, 9, 43300–43314. 10.1021/acsami.7b06295. 29140074

[ref54] Ouyang L.; Qi M.; Wang S.; Tu S.; Li B.; Deng Y.; Yang W. Osteogenesis and antibacterial activity of graphene oxide and dexamethasone coatings on porous polyetheretherketone via polydopamine-assisted chemistry. Coatings 2018, 8, 203 10.3390/coatings8060203.

[ref55] Fernandez-Yague M. A.; Larrañaga A.; Gladkovskaya O.; Stanley A.; Tadayyon G.; Guo Y.; Sarasua J. R.; Tofail S. A.; Zeugolis D. I.; Pandit A.; Biggs M. J. Effects of Polydopamine Functionalization on Boron Nitride Nanotube Dispersion and Cytocompatibility. Bioconjugate Chem. 2015, 26, 2025–2037. 10.1021/acs.bioconjchem.5b00257. 26282841

[ref56] Ryu J. H.; Messersmith P. B.; Lee H. Polydopamine Surface Chemistry: A Decade of Discovery. ACS Appl. Mater. Interfaces 2018, 10, 7523–7540. 10.1021/acsami.7b19865. 29465221 PMC6320233

[ref57] Thakur V. K.; Yan J.; Lin M. F.; Zhi C.; Golberg D.; Bando Y.; Sim R.; Lee P. S. Novel polymer nanocomposites from bioinspired green aqueous functionalization of BNNTs. Polym. Chem. 2012, 3, 962–969. 10.1039/c2py00612j.

[ref58] Wu H.; Kessler M. R. Multifunctional cyanate ester nanocomposites reinforced by hexagonal boron nitride after noncovalent biomimetic functionalization. ACS Appl. Mater. Interfaces 2015, 7, 5915–5926. 10.1021/acsami.5b00147. 25726956

[ref59] Michalicha A.; Pałka K.; Roguska A.; Pisarek M.; Belcarz A. Polydopamine-coated Curdlan Hydrogel as A Potential Carrier of Free Amino Group-containing Molecules. Carbohydr. Polym. 2021, 256, 117524 10.1016/j.carbpol.2020.117524. 33483045

[ref60] Lim K.; Chua R. R. Y.; Ho B.; Tambyah P. A.; Hadinoto K.; Leong S. S. J. Development of A Catheter Functionalized by A Polydopamine Peptide Coating with Antimicrobial and Antibiofilm Properties. Acta Biomater. 2015, 15, 127–138. 10.1016/j.actbio.2014.12.015. 25541344

[ref61] Singh I.; Priyam A.; Jha D.; Dhawan G.; Gautam H. K.; Kumar P. Polydopamine-aminoglycoside Nanoconjugates: Synthesis, Characterization, Antimicrobial Evaluation and Cytocompatibility. Mater. Sci. Eng.,C 2020, 107, 110284 10.1016/j.msec.2019.110284. 31761233

[ref62] Batul R.; Bhave M.; J Mahon P.; Yu A.; Yu A. Polydopamine Nanosphere with In-situ Loaded Gentamicin and Its Antimicrobial Activity. Molecules 2020, 25, 2090 10.3390/molecules25092090. 32365745 PMC7250025

[ref63] Tam V. H.; Kabbara S.; Vo G.; Schilling A. N.; Coyle E. A. Comparative pharmacodynamics of gentamicin against Staphylococcus aureus and Pseudomonas aeruginosa. Antimicrob. Agents Chemother. 2006, 50, 2626–2631. 10.1128/aac.01165-05. 16870751 PMC1538660

[ref64] Hayward R. S.; Harding J.; Molloy R.; Land L.; Longcroft-Neal K.; Moore D.; Ross J. D. Adverse effects of a single dose of gentamicin in adults: a systematic review. Br. J. Clin. Pharmacol. 2018, 84, 223–238. 10.1111/bcp.13439. 28940715 PMC5777443

[ref65] Hu J.; Yang L.; Cheng X.; Li Y.; Cheng Y. Aminoglycoside-based biomaterials: from material design to antibacterial and gene delivery applications. Adv. Funct. Mater. 2021, 31, 2103718 10.1002/adfm.202103718.

[ref66] Ren X.; van der Mei H. C.; Ren Y.; Busscher H. J.; Peterson B. W. Antimicrobial loading of nanotubular titanium surfaces favoring surface coverage by mammalian cells over bacterial colonization. Mater. Sci. Eng.,C 2021, 123, 112021 10.1016/j.msec.2021.112021. 33812638

[ref67] Zhang J.; Singh P.; Cao Z.; Rahimi S.; Pandit S.; Mijakovic I. Polydopamine/graphene oxide coatings loaded with tetracycline and green Ag nanoparticles for effective prevention of biofilms. Appl. Surf. Sci. 2023, 626, 157221 10.1016/j.apsusc.2023.157221.

[ref68] Song J.; Dai Z.; Li J.; Tong X.; Zhao H. Polydopamine-decorated Boron Nitride as Nano-reinforcing Fillers for Epoxy Resin with Enhanced Thermomechanical and Tribological Properties. Mater. Res. Express 2018, 5, 075029 10.1088/2053-1591/aab529.

[ref69] Quan K.; Jiang G.; Liu J.; Zhang Z.; Ren Y.; Busscher H. J.; van der Mei H. C.; Peterson B. W. Influence of interaction between surface-modified magnetic nanoparticles with infectious biofilm components in artificial channel digging and biofilm eradication by antibiotics in vitro and in vivo. Nanoscale 2021, 13, 4644–4653. 10.1039/d0nr08537e. 33616592

[ref70] Sheng M.; Yang R.; Gong H.; Zhang Y.; Lin X.; Jing J. Enhanced thermal conductivity and stability of boron nitride/phenyl silicone rubber composites via surface modification and grain alignment. J. Mater. Sci. 2022, 57, 5805–5824. 10.1007/s10853-021-06860-8.

[ref71] Tarhan T.; Şen Ö.; Ciofani M. E.; Yılmaz D.; Çulha M. Synthesis and characterization of silver nanoparticles decorated polydopamine coated hexagonal boron nitride and its effect on wound healing. J. Trace Elem. Med. Biol. 2021, 67, 126774 10.1016/j.jtemb.2021.126774. 33984543

[ref72] Songfeng E.; Ye X.; Wang M.; Huang J.; Ma Q.; Jin Z.; Ning D.; Lu Z. Enhancing the Tribological Properties of Boron Nitride by Bioinspired Polydopamine Modification. Appl. Surf. Sci. 2020, 529, 147054 10.1016/j.apsusc.2020.147054.

[ref73] Wu H.; Kessler M. R. Multifunctional Cyanate Ester Nanocomposites Reinforced by Hexagonal Boron Nitride after Noncovalent Biomimetic Functionalization. ACS Appl. Mater. Interfaces 2015, 7, 5915–5926. 10.1021/acsami.5b00147. 25726956

[ref74] Ding D.; Shang Z.; Zhang X.; Lei X.; Liu Z.; Zhang Q.; Chen Y. Greatly Enhanced Thermal Conductivity of Polyimide Composites by Polydopamine Modification and the 2D-aligned Structure. Ceram. Int. 2020, 46, 28363–28372. 10.1016/j.ceramint.2020.07.340.

[ref75] Zhao M.; Deng C.; Zhang X. The Design and Synthesis of A Hydrophilic Core-shell-shell Structured Magnetic Metal-Organic Framework as A Novel Immobilized Metal Ion Affinity Platform for Phosphoproteome Research. Chem. Commun. 2014, 50, 6228–6231. 10.1039/c4cc01038h. 24789051

[ref76] Zangmeister R. A.; Morris T. A.; Tarlov M. J. Characterization of Polydopamine Thin Films Deposited at Short Times by Autoxidation of Dopamine. Langmuir 2013, 29, 8619–8628. 10.1021/la400587j. 23750451

[ref77] Feng Y.; Ma X.; Chang L.; Zhu S.; Guan S. Characterization and Cytocompatibility of Polydopamine on MAO-HA Coating Supported on Mg-Zn-Ca Alloy. Surf. Interface Anal. 2017, 49, 1115–1123. 10.1002/sia.6286.

[ref78] Alhariri M.; Majrashi M. A.; Bahkali A. H.; Almajed F. S.; Azghani A. O.; Khiyami M. A.; Alyamani E. J.; Aljohani S. M.; Halwani M. A. Efficacy of Neutral and Negatively Charged Liposome-loaded Gentamicin on Planktonic Bacteria and Biofilm Communities. Int. J. Nanomed. 2017, 12, 6949–6961. 10.2147/ijn.s141709. PMC5609801 29075113

[ref79] Lebeaux D.; Ghigo J. M.; Beloin C. Biofilm-related Infections: Bridging the Gap Between Clinical Management and Fundamental Aspects of Recalcitrance Toward Antibiotics. Microbiol. Mol. Biol. Rev. 2014, 78, 510–543. 10.1128/mmbr.00013-14. 25184564 PMC4187679

[ref80] Ran H. H.; Cheng X.; Gao G.; Sun W.; Jiang Y. W.; Zhang X.; Jia H. R.; Qiao Y.; Wu F. G. Colistin-loaded Polydopamine Nanospheres Uniformly Decorated with Silver Nanodots: A Nanohybrid Platform with Improved Antibacterial and Antibiofilm Performance. ACS Appl. Bio Mater. 2020, 3, 2438–2448. 10.1021/acsabm.0c00163. 35025293

[ref81] Yu N.; Wang X.; Qiu L.; Cai T.; Jiang C.; Sun Y.; Li Y.; Peng H.; Xiong H. Bacteria-triggered Hyaluronan/AgNPs/gentamicin Nanocarrier for Synergistic Bacteria Disinfection and Wound Healing Application. Chem. Eng. J. 2020, 380, 122582 10.1016/j.cej.2019.122582.

[ref82] Borovinskaya M. A.; Pai R. D.; Zhang W.; Schuwirth B. S.; Holton J. M.; Hirokawa G.; Kaji H.; Kaji A.; Cate J. H. D. Structural Basis for Aminoglycoside Inhibition of Bacterial Ribosome Recycling. Nat. Struct. Mol. Biol. 2007, 14, 727–732. 10.1038/nsmb1271. 17660832

[ref83] Liu J.; Wang Y.; Ma J.; Peng Y.; Wang A. A review on bidirectional analogies between the photocatalysis and antibacterial properties of ZnO. J. Alloys Compd. 2019, 783, 898–918. 10.1016/j.jallcom.2018.12.330.

[ref84] Ramalingam B.; Parandhaman T.; Das S. K. Antibacterial effects of biosynthesized silver nanoparticles on surface ultrastructure and nanomechanical properties of gram-negative bacteria viz. Escherichia coli and Pseudomonas aeruginosa. ACS Appl. Mater. Interfaces 2016, 8, 4963–4976. 10.1021/acsami.6b00161. 26829373

[ref85] Parandhaman T.; Das S. K. Facile synthesis, biofilm disruption properties and biocompatibility study of a poly-cationic peptide functionalized graphene–silver nanocomposite. Biomater. Sci. 2018, 6, 3356–3372. 10.1039/c8bm01003j. 30357139

[ref86] Wang L.; Hu C.; Shao L. The antimicrobial activity of nanoparticles: present situation and prospects for the future. Int. J. Nanomed. 2017, 12, 1227–1249. 10.2147/ijn.s121956. PMC5317269 28243086

[ref87] Carlson C.; Hussain S. M.; Schrand A. M.; K Braydich-Stolle L.; Hess K. L.; Jones R. L.; Schlager J. J.; Schlager J. J. Unique cellular interaction of silver nanoparticles: size-dependent generation of reactive oxygen species. J. Phys. Chem. B 2008, 112, 13608–13619. 10.1021/jp712087m. 18831567

[ref88] Kakarla A. B.; Kong I. In vitro and in vivo cytotoxicity of boron nitride nanotubes: a systematic review. Nanomaterials 2022, 12, 2069 10.3390/nano12122069. 35745407 PMC9229602

[ref89] Xin X.; Barger M.; Roach K. A.; Bowers L.; Stefaniak A. B.; Kodali V.; Glassford E.; Dunn K. L.; Dunn K. H.; Wolfarth M.; Friend S.; et al. Toxicity evaluation following pulmonary exposure to an as-manufactured dispersed boron nitride nanotube (BNNT) material in vivo. NanoImpact 2020, 19, 100235 10.1016/j.impact.2020.100235.

[ref90] Kodali V. K.; Roberts J. R.; Shoeb M.; Wolfarth M. G.; Bishop L.; Eye T.; Barger M.; Roach K. A.; Friend S.; Schwegler-Berry D.; Chen B. T.; et al. Acute in vitro and in vivo toxicity of a commercial grade boron nitride nanotube mixture. Nanotoxicology 2017, 11, 1040–1058. 10.1080/17435390.2017.1390177. 29094619

[ref91] Soares D. C. F.; Ferreira T. H.; Ferreira C. d. A.; Cardoso V. N.; de Sousa E. M. B. Boron nitride nanotubes radiolabeled with 99mTc: Preparation, physicochemical characterization, biodistribution study, and scintigraphic imaging in Swiss mice. Int. J. Pharm. 2012, 423, 489–495. 10.1016/j.ijpharm.2011.12.002. 22178127

