# Supplementary material for: Antibiotic-Loaded Boron Nitride Nanoconjugate with Strong Performance against Planktonic Bacteria and Biofilms
Source: ACS Appl Bio Mater. 2023 Jul 20;6(8):3131–42. doi: 10.1021/acsabm.3c00247 (PMC10445265; doi:10.1021/acsabm.3c00247)
Supplement: Supplementary file 1 — mt3c00247_si_001.pdf [file mt3c00247_si_001.pdf]

Antibiotic-loaded Boron Nitride Nanoconjugate with Strong Performance against Planktonic Bacteria and Biofilms

Jian Zhang[1#], Nisha Neupane[1,2#], Puspa Raj Dahal[2], Shadi Rahimi[1], Zhejian Cao[1], Santosh Pandit[1*], Ivan Mijakovic[1,3*]

1 Systems and Synthetic Biology Division, Department of Life Sciences, Chalmers University of Technology, SE-412 96 Gothenburg, Sweden

2 Department of Microbiology, Tri-Chandra Multiple College, Tribhuvan University, Kathmandu, 46000, Nepal

3 The Novo Nordisk Foundation, Center for Biosustainability, Technical University of Denmark, DK-2800 Kogens Lyngby, Denmark

# Authors contributed equally to this work. * Corresponding authors.

Santosh Pandit: pandit@chalmers.se; Ivan Mijakovic: ivan.mijakovic@chalmers.se

Figure S1. SEM image of BN, PBN and GPBN and EDS elemental mapping of GPBN.

Table S1. Hydrodynamic size and zeta potential of the BN, PBN and GPBN.

Table S2. Surface elemental composition of BN, PBN and GPBN.

Figure S2. Raman spectra of BN, PBN and GPBN.

Figure S3. FTIR spectra of BN, PBN and GPBN.

Figure S4. Amount of gentamicin loading and release for GPBN.

Figure S5. Area ration of dead/live cells of S. aureus and E. Coli biofilms after exposure of GPBN.

Figure S6. SEM images of $GPBN_{250}$ (a, d), $GPBN_{500}$ (b, e) and $GPBN_{1000}$ (c, f) coatings.

Figure S7. AlamarBlue cell viability 24 h after administration of BN (a), PBN (b) and GPBN (c).

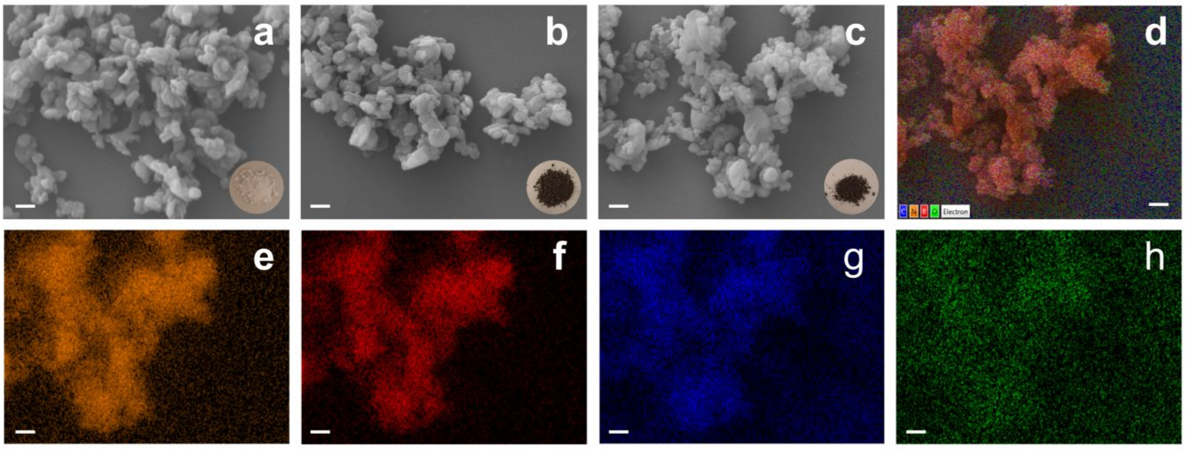

Figure S1. SEM image of BN (a), PBN (b) and GPBN (c); powder of BN, PBN, GPBN is shown in the bottom right inset of figures; EDS elemental mapping (d) and C, B, N, O mapping of GPBN (e-h).

	Size (nm)	Zeta potential (mV)
BN	176.5 ± 9.3	-36.6 ± 0.4
PBN	193.6 ± 10.5	-19.8 ± 0.5
GPBN	220.8 ± 13.1	+ 6.2 ± 0.3

Table S1. Hydrodynamic size and zeta potential of the BN, PBN and GPBN.

Nanoconjugates	Atomic percentages			
	B1s (%)	N1s (%)	C1s (%)	O1s (%)
BN	46.08	45.53	3.84	4.56
PBN	9.38	14.08	57.06	19.48
GPBN	9.28	14.56	55.64	20.52

Table S2. Surface elemental composition of BN, PBN and GPBN.

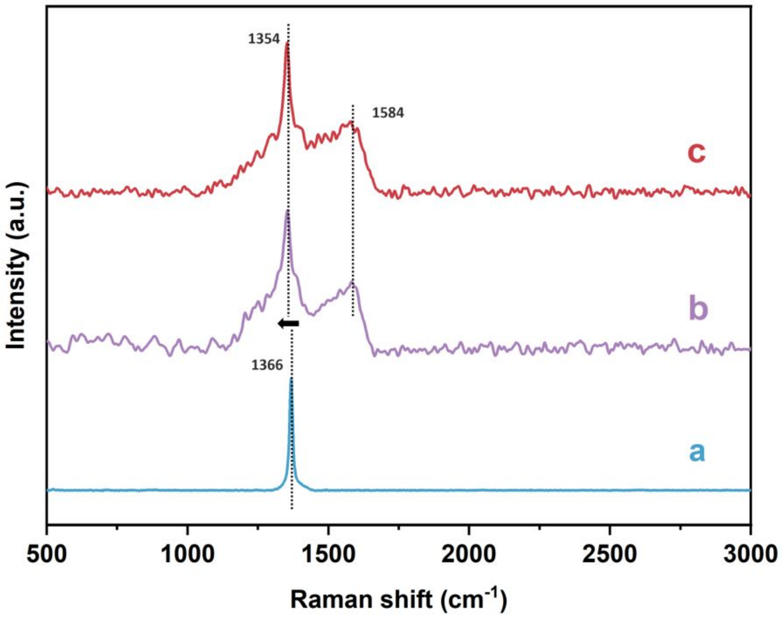

Figure S2. Raman spectra of BN (a), PBN (b) and GPBN (c).

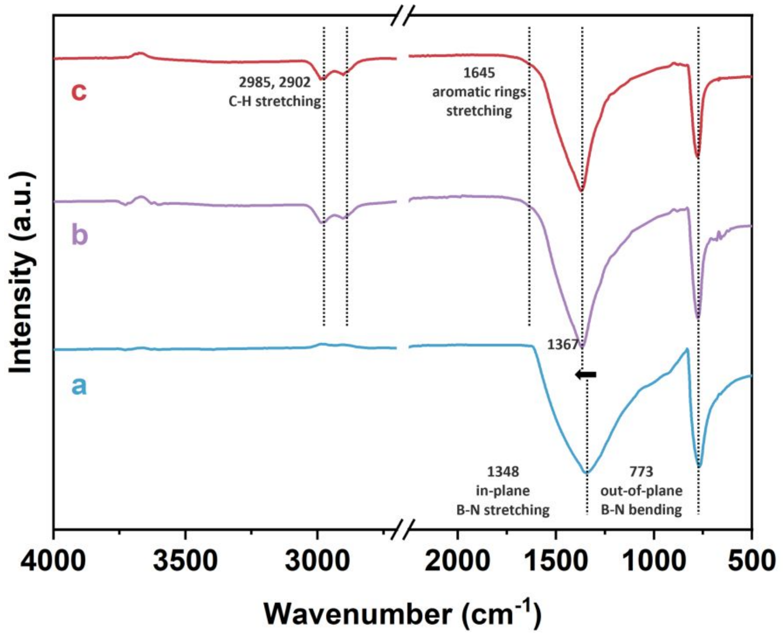

Figure S3. FTIR spectra of BN (a), PBN (b) and GPBN (c).

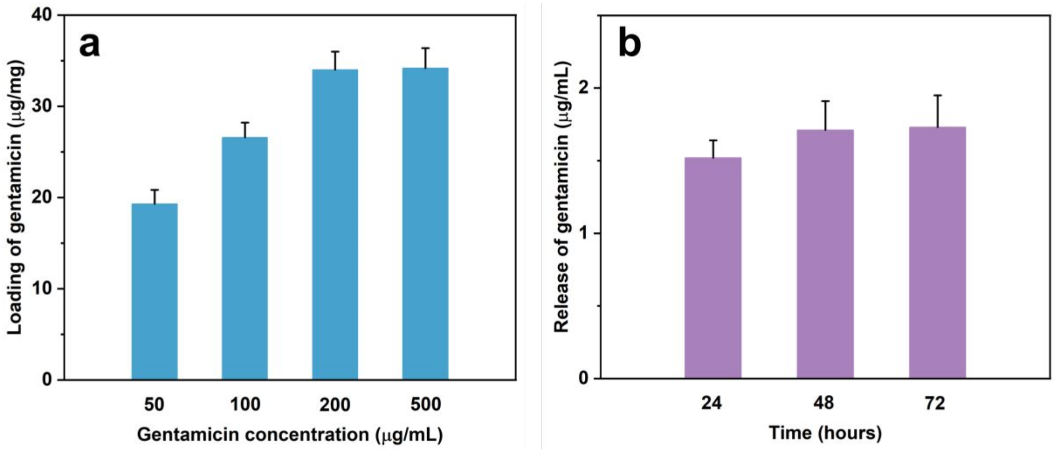

Figure S4. (a) The amount of gentamicin loaded on PBN at different concentration. (b) The cumulative amount of gentamicin released from GPBN after immersion in pH 7.4 PBS buffer.

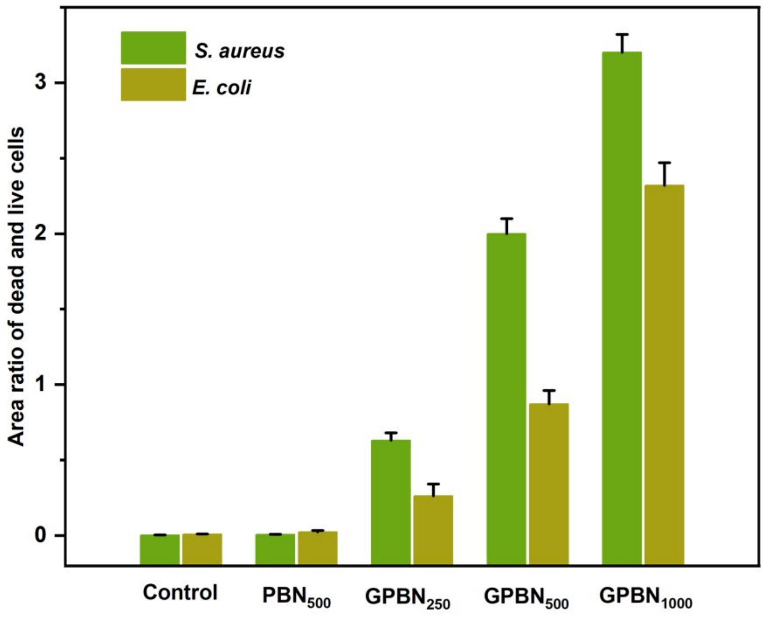

Figure S5. Area ration of dead/live cells of *S. aureus* and *E. Coli* biofilms after exposure of GPBN.

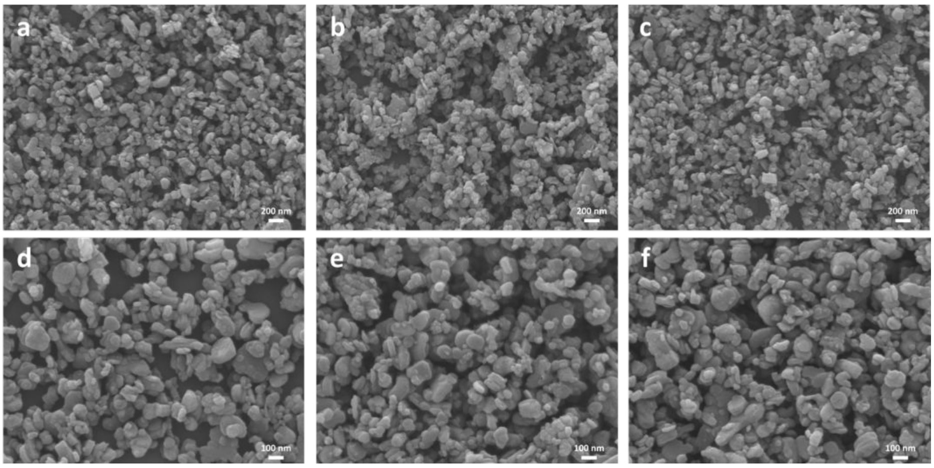

Figure S6. SEM images of $GPBN_{250}$ (a, d), $GPBN_{500}$ (b, e) and $GPBN_{1000}$ (c, f) coatings.

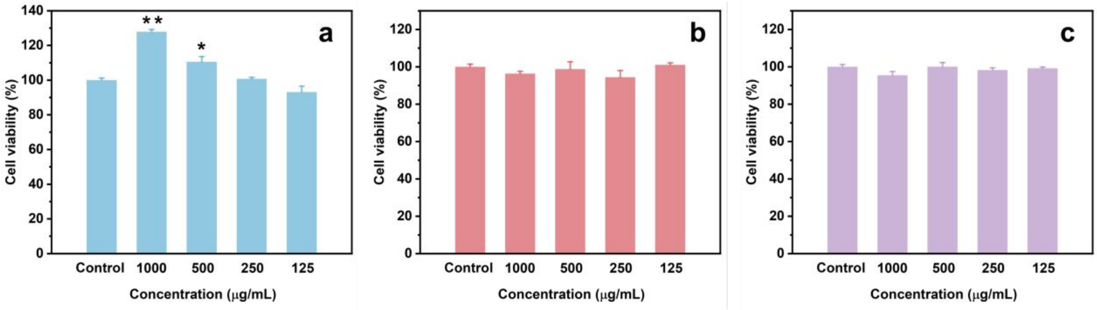

Figure S7. AlamarBlue cell viability assay 24 h after administration of the BN (a), PBN (b) and GPBN (c). All values are normalized to those obtained for untreated cells (medium alone).
